# QTL Mapping of Seed Quality Traits Including Cooking Time, Flavor, and Texture in a Yellow Dry Bean (*Phaseolus vulgaris* L.) Population

**DOI:** 10.3389/fpls.2021.670284

**Published:** 2021-06-22

**Authors:** Amber Bassett, Dennis N. Katuuramu, Qijian Song, Karen Cichy

**Affiliations:** ^1^Department of Plant, Soil and Microbial Sciences, Michigan State University, East Lansing, MI, United States; ^2^U.S. Vegetable Laboratory, USDA-ARS, Charleston, SC, United States; ^3^Beltsville Agricultural Research Center, USDA-ARS, Beltsville, MD, United States; ^4^Sugarbeet and Bean Research Unit, USDA-ARS, East Lansing, MI, United States

**Keywords:** sensory quality, taste, legume, fast cooking, Manteca

## Abstract

Manteca yellow dry beans (*Phaseolus vulgaris* L.) have many quality traits that appeal to consumers, including fast cooking times, creamy texture, and sweet, buttery flavor. They are native to Chile and consumed in regions of South America and Africa but are largely unfamiliar to United States consumers. While cooking time, flavor, and texture have not been prioritized in United States dry bean breeding programs, genetic variability exists such that these traits could be addressed through breeding. In this study, a recombinant inbred line (RIL) population was developed from a cross between Ervilha (Manteca) and PI527538 (Njano), yellow dry beans with contrasting cooking time and sensory attributes. The population and parents were grown for 2 years in Michigan and evaluated for cooking time and sensory attribute intensities, including total flavor, beany, vegetative, earthy, starchy, sweet, bitter, seed-coat perception, and cotyledon texture. Cooking time ranged 19–34 min and exhibited high broad-sense heritability (0.68). Sensory attribute intensities also exhibited variation among RILs, although broad-sense heritability was low, with beany and total flavor exhibiting the highest (0.33 and 0.27). A linkage map of 870 single nucleotide polymorphisms markers across 11 chromosomes was developed for quantitative trait loci (QTL) mapping, which revealed QTL for water uptake (3), cooking time (6), sensory attribute intensities (28), color (13), seed-coat postharvest non-darkening (1), seed weight (5), and seed yield (2) identified from data across 2 years. Co-localization was identified for starchy, sweet, and seed-coat perception on Pv01; for total flavor, beany, earthy, starchy, sweet, bitter, seed-coat perception, cotyledon texture, and color on Pv03; water uptake and color on Pv04; total flavor, vegetative, sweet, and cotyledon texture on Pv07; cooking time, starchy, sweet, and color on Pv08; and water uptake, cooking time, total flavor, beany, starchy, bitter, seed-coat perception, cotyledon texture, color, and seed-coat postharvest non-darkening on Pv10. The QTL identified in this work, in particular CT8.2 and CT10.2, can be used to develop molecular markers to improve seed quality traits in future dry bean varieties. Considering yellow dry beans already excel in quality and convenience, they might be an ideal market class to signal a new focus on consumer-valued traits in the United States.

## Introduction

Dry beans (*Phaseolus vulgaris* L.) are widely regarded as a nutritious and affordable food (Akibode and Maredia, 2011). The species encompasses many different market classes grown and consumed around the world with many regional preferences (Siddiq and Uebersax, 2012). There is variability not just for seed size, color, and shape, but also end-use quality attributes, including cooking time, mineral concentration and bioavailability, color, flavor, and texture (Katuuramu et al., 2018; Bassett et al., 2020b). Some market classes may be of particular interest to modern consumers looking to incorporate beans into their diets for their nutritional benefits and also looking for convenience not typically associated with dry beans considering their often long cooking times (Sloan, 2015).

The Manteca yellow bean market class has multiple quality traits of value to consumers (Leakey, 2000; Wiesinger et al., 2016, 2018). Manteca are pale yellow with a gray hilum. They are Andean beans native to Chile (Leakey, 1992) and currently consumed in South America and Africa (Wiesinger et al., 2018). Manteca are appreciated for their sweet, buttery flavor (Leakey, 2000) as well as fast cooking time and high iron bioavailability (Wiesinger et al., 2016, 2018). United States consumers are largely unfamiliar with this yellow market class. Its novel color has the potential to set the market class apart from familiar market classes and signal the presence of quality attributes if introduced more broadly.

Current dietary guidelines recommend $\frac{1}{4}$ cup (∼56 g) of pulse per day, but less than 50% of the population meets that recommendation (Britten et al., 2012). There is an opportunity to increase utilization of dry beans by addressing consumer preferences for convenience and flavor as well as developing bean products to reach new consumers (IPSOS, 2010; Karlsen et al., 2016; Hooper et al., 2019; Winham et al., 2019). While United States dry bean breeders have always prioritized quality traits, they primarily have focused on seed size, shape, color, and canning quality and production-related traits with minor if any consideration for cooking time and flavor (Kelly and Cichy, 2012). As a result, genetic variability exists for cooking time, flavor, and texture in modern cultivars as well as the breeding lines used for their development (Bassett et al., 2020b). There is an opportunity to address these consumer-valued traits through breeding to increase dry bean consumption, and Manteca beans are an ideal target for this effort, as they already excel in these traits and provide novelty to those unfamiliar with them.

Cooking time has been reported to be controlled by few genes and have moderate to high heritability, with narrow sense heritability values estimated between 0.74 and 0.90 (Elia et al., 1997; Jacinto-Hernandez et al., 2003). Genotypic cooking time patterns are stable across environments (Cichy et al., 2019; Katuuramu et al., 2020). Following screening of 206 accessions of the Andean Diversity Panel (ADP), several significant single nucleotide polymorphisms (SNPs) associated with cooking time were identified on Pv02, Pv03, and Pv06 (Cichy et al., 2015b). A more recent screening of 430 accessions of the ADP revealed additional significant SNPs on Pv03, Pv04, Pv06, Pv07, Pv08, and Pv11 (Bassett et al., 2020b). In addition, a recent quantitative trait loci (QTL) mapping study using a recombinant inbred line (RIL) population developed from two ADP accessions revealed QTL for cooking time on Pv01, Pv02, Pv03, Pv05, Pv06, Pv10, and Pv11 (Berry et al., 2020). With further study, marker-assisted selection may be a feasible method for breeding faster cooking beans, which could reduce the need to phenotype for cooking time and allow greater incorporation of the fast-cooking trait in breeding programs.

Flavor is a major influence on consumer food choices (Glanz et al., 1998), but evaluating flavor and texture is time consuming and requires trained panelists. As it stands, little is understood about consumer preference in regard to flavor and texture in dry beans apart from a general preference for beans that are sweet and soft and for bean products without a beany “off” flavor (Kinsella, 1979; Bott and Chambers, 2006; Mkanda et al., 2007; Hooper et al., 2019). A few studies have identified genetic variability for sensory attributes, including flavor and texture acceptability, seed-coat perception, seed-coat roughness, cotyledon mealiness, and beany flavor intensity (Koehler et al., 1987; Rivera et al., 2013). A recent study identified genetic variability in the Andean Diversity Panel (ADP) for total, beany, vegetative, earthy, starchy, bitter, and sweet flavor intensities as well as seed-coat perception and cotyledon texture (Bassett et al., 2020b). Using a genome-wide association approach, significant SNPs were identified for many of these traits. As for cooking time, the potential for marker-assisted selection could reduce the need for extensive phenotyping and allow breeders to incorporate flavor and texture into their breeding programs more easily. With a greater understanding of consumer preference for flavor and texture, new varieties could be developed that appeal to consumers and are suitable for use as ingredients in products.

In this study, a yellow dry bean RIL population developed from two ADP accessions with contrasting cooking time and sensory characteristics was screened for cooking time and sensory attribute intensities to elucidate their genetic control and aid in the development of molecular markers for these traits.

## Materials and Methods

### Germplasm

**FIGURE 1:**
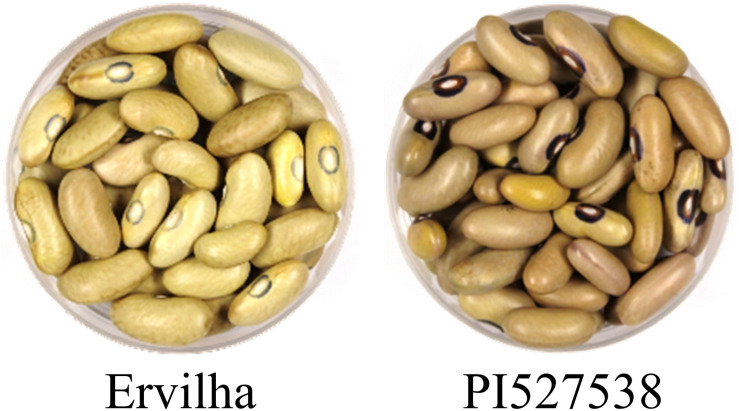
Images of Ervilha and PI527538 raw seeds.

A RIL population of 242 F_5_:F_7_-F_8_ lines was developed from a cross between two yellow bean genotypes of the Andean gene pool: Ervilha (ADP0512) and PI527538 (ADP0468) (Figure 1; Bassett and Cichy, 2020). The RILs were developed by advancing F_2_ seed *via* single seed descent to the F_5_ generation and then bulking seeds from individual plants to form RILs.

Ervilha is a pale yellow Manteca seed type with a gray hilum with a seed weight of 52.8 (g per 100 seeds) in this study (Supplementary Table 1). Ervilha was originally collected at a marketplace in Angola in 2010 (Cichy et al., 2015a). PI527538 is a yellow-green Njano seed type with hints of purple and a black hilum with a seed weight of 48.0 (g per 100 seeds) in this study (Supplementary Table 1). PI527538 was originally collected in Burundi in 1985 (Cichy et al., 2015a). Both genotypes are likely members of race Nueva Granada. These genotypes were selected to develop a RIL population after a screening of 206 lines of the Andean Diversity Panel (ADP) for cooking time, mineral concentration, and iron bioavailability (Cichy et al., 2015b; Katuuramu et al., 2018). Ervilha cooks about 10 min faster than PI527538 when soaked, and this relative difference in cooking time is stable across growing environments, although specific cooking times vary depending on the growing environment and storage conditions (Cichy et al., 2015b; Katuuramu et al., 2020).

The genotypes were grown at the Montcalm Research Farm in Lakeview, MI in 2016 and 2017. The soil type is Eutric Glossoboralfs (coarse-loamy and mixed) and Alfic Fragiorthods (coarse-loamy, mixed, and frigid). Two row plots 4.75 m long with 0.5 m spacing between rows were arranged in a randomized complete block design with two replications per genotype. In 2016, 100 seeds were planted per plot due to limited seed, and in 2017, 160 seeds were planted per plot. Standard agronomic practices were followed as described in the MSU SVREC 2017 Farm Research Report (Kelly et al., 2017). Plants were hand-pulled at maturity and threshed with a Hege 140 plot harvester (Wintersteiger, Salt Lake City, UT, United States). Following harvest, seeds were cleaned by hand to remove field debris, off types, and damaged seed. Seed weights (g per 100 seeds) and seed yield (kg per ha) were recorded for each field replicate.

### CIELAB Analysis and Seed-Coat Postharvest Darkening

For both years, images were collected for one field replicate of each genotype using a custom machine vision system as described in Mendoza et al. (2017). For each image, a 60 × 15 mm petri dish was filled with representative seeds cleaned of debris and damaged seeds. The EOS Rebel T3i software settings were consistent across each image as follows: lens aperture *f* = 5.6, shutter speed 1/125, white balanced, and ISO = 100. Following image collection, each image was cropped to center the petri dish and minimize background. To examine the relationship among color, cooking time, and sensory attributes, CIELAB values were obtained using a custom batch macro in ImageJ developed by Bornowski et al. (2020) that applies a gamma correction of 0.5, excludes background pixels outside the petri dish, and measures each slice of the LAB stack. CIELAB uses three values to describe color: L^∗^ for black (0) to white (100), a^∗^ for green (−) to red (+), and b^∗^ for blue (−) to yellow (+). These values were collected relative to the imaging conditions and reflect average color of seeds without calibration for the purpose of observing differences among lines rather than determining absolute color.

Variability in seed-coat postharvest darkening among genotypes was observed after the first year, so the potential presence of the non-darkening trait in this population was explored. Genotypes grown in 2017 were stored for approximately 2 years in opaque paper bags in a cool, dry barn prior to evaluation for seed-coat postharvest darkening in January 2020. Samples that appeared visibly darkened after this storage period were given a score of 1 and those that remained light were given a score of 0.

### Cooking Time Evaluation

For each year, two field replicates of 30 seed per genotype were equilibrated to 10–14% moisture in a 4°C humidity chamber prior to evaluating for cooking time. Each 30 seed sample was soaked for 12 h in distilled water prior to cooking time evaluation using an automated Mattson cooker method (Wang and Daun, 2005). Genotypes were cooked in a random order to minimize seed aging effects. Seed weights after soaking were recorded for each sample to determine water uptake. Mattson cookers loaded with soaked seeds were placed into 4 L stainless steel beakers with 1.8 L of boiling distilled water on Cuisinart CB-30 Countertop Single Burners to cook. The Mattson cookers (Michigan State University Machine Shop, East Lansing, MI, United States) use twenty-five 65 g stainless steel rods with 2 mm diameter pins to pierce beans as they finish cooking in each well. As the pins drop, a custom software reports the cooking time associated with each pin. A low boil was maintained during cooking, and the 80% cooking times were recorded and regarded as the time required to fully cook each sample. Final cooked seed weights were recorded.

### Sensory Evaluation

Ervilha, PI527538, and the RILs were evaluated in duplicate using a modified Quantitative Descriptive Analysis (QDA) approach (Stone et al., 1974), in which four panelists per session independently evaluated samples using a non-consensus approach to limit group bias. For the purposes of this study, the QDA approach was modified as described by Bassett et al. (2020b) to increase suitability for implementation in public breeding programs with limited resources. In brief, seeds from each field replicate were prepared for sensory evaluation in the same order that they were cooked for cooking time evaluation to minimize seed aging effects. Sensory evaluation sessions were held daily with four panelists per session until each genotype had been evaluated twice for each year. Twelve genotypes were evaluated at each session including Ervilha and PI527538 as controls. Each sample was evaluated using 5-point attribute intensity scales (low → high intensity) for total, beany, vegetative, earthy, starchy, bitter, and sweet flavor intensities as well as seed-coat perception and cotyledon texture. The scale for seed-coat perception ranged from imperceptible (1) to tough and lingering (5). For cotyledon texture, the scale ranged from mushy (1) to very gritty/firm (5) (Bassett et al., 2020b). This sensory evaluation protocol was approved by the Institutional Review Board of Michigan State University (IRB# x16-763e Category: Exempt 6).

### Panel Training

Panelists were recruited from the USDA (East Lansing, MI, United States) and Michigan State University dry bean breeding programs due to their familiarity with dry beans and their availability for long-term sensory evaluation projects. Initially, seven panelists were trained using a diverse set of dry bean genotypes selected from the USDA and MSU dry bean programs with the intention of exposing panelists to a wide range of attribute intensities. This initial set included dark red kidney, Jacob’s cattle, white kidney, and yellow beans. A training set of genotypes exhibiting extreme attribute intensities identified in the ADP (Bassett et al., 2020b) was used to train eleven panelists for the second year. This training set was grown at the MSU Montcalm Research Center in Lakeview, MI alongside the RIL population.

Panelists were trained over multiple sessions using a consensus approach. Panelists then practiced using a non-consensus approach to improve their familiarity with the selected scales and their sensory evaluation skills. Panelist performance was assessed *via* ANOVA with F_Genotype_ (*p*-value < 0.05) indicating ability to discriminate and F_rep_ (*p*-value > 0.05) indicating consistency (Meilgaard et al., 1999; Armelim et al., 2006). Sensory evaluation commenced after successful training of each panelist. Following screening of the parents and RILs from both years, panel performance was assessed as during training.

### Sample Preparation for Sensory Evaluation

Samples were prepared as described in Bassett et al. (2020b). Prior to each session, four seeds per panelist of each genotype scheduled for evaluation were soaked for 12 h in distilled water prior to cooking. Large tea bags filled with the soaked samples were boiled in distilled water for the cooking time determined by the Mattson cooker method, timed so they all finished cooking together. The cooked samples were poured into preheated (105°C) ceramic ramekins, covered with aluminum foil, and placed in a chafing dish to maintain temperature prior to evaluation. Samples were given a random letter code to mask their identity. Panelists were asked to refrain from wearing strong scents or eating during the hour before each session. Samples were served out of the ceramic ramekins with a plastic spoon onto paper plates. Lemon water was made available as a palate cleanser, and panelists were asked to drink water between samples.

### Statistics

PROC MIXED in SAS version 9.4 of the SAS System for Windows (SAS Institute Inc., Cary, NC, United States) was used to conduct analyses of variance (ANOVAs) for all traits. For seed weight, seed yield, water uptake, and cooking time, the fixed effects were genotype, year, and genotype by year with replicate as a random effect. For L^∗^, a^∗^, and b^∗^ color values, the fixed effects were genotype and year with no random effects. For the sensory attribute intensity traits, the fixed effects were genotype, year, and genotype by year with replicate, panelist (year), and session (year) as random effects. Least squares estimates for sensory traits were calculated *via* the LSMeans statement in PROC MIXED for visualization of trait distributions. Mean separation of parents was determined using pdiff in PROC MIXED. Density plots of traits were generated in R (R Core Team, 2017) using the sm package version 2.2–5.6 (Bowman and Azzalini, 2018).

To analyze both years combined while minimizing environmental effects, best linear unbiased predictors (BLUPs) were generated for each trait using the lme4 package (Bates et al., 2015) in R version 4.0.3 with genotype, year, genotype by year, and rep nested in year as random effects. For sensory traits, panelist nested in year and session nested in year were also included as random effects. For analysis within individual years, BLUPs were calculated for sensory traits with genotype, rep, panelist, and session included as random effects. The normality of trait distributions was assessed visually using Q–Q plots.

Broad sense heritability (H^2^) was calculated on a family mean basis for each trait using the following equation:

$$H^{2}=\frac{\sigma_{g}^{2}}{\sigma_{g}^{2}+\frac{\sigma_{g\,y}^{2}}{y}+\frac{\sigma_{e}^{2}}{y\,r}}$$

where $\sigma_{g}^{2}$ is genotypic variance, $\sigma_{g\,y}^{2}$ is genotype year interaction variance, $\sigma_{e}^{2}$ is environmental variance, *y* is number of years, and *r* is number of replications. For seed-coat postharvest non-darkening, the $\frac{\sigma_{g\,y}^{2}}{y}$ component was excluded from the equation as the trait was only assessed in 1 year. Variance components were calculated using PROC VARCOMP in SAS version 9.4 with method = restricted maximum likelihood method (reml) (Holland et al., 2003). Principal component analysis (PCA) among traits was conducted *via* singular value decomposition of the centered and scaled BLUPs from both years combined using the prcomp function in R.

### Genotyping

Genomic DNA was extracted from young trifoliate leaf tissue from three plants each for the 242 RILs and the two parental lines (Ervilha and PI527538) using a Macherey-Nagel NucleoSpin Plant II kit. For each genotype, 10 μL aliquots of DNA at a minimum concentration of 50 ng/μL were loaded into 96-well plates with parental lines prepared in quintuplicate. The plates were genotyped at the USDA Beltsville Agricultural Research Center in Beltsville, MD (BARC) using the BARCBean12K BeadChip, which includes all SNPs from the BARCBean6K_3 (Song et al., 2015) and additional SNPs among a set of Andean accessions. Illumina’s GenomeStudio software was used to confirm variant calling. SNP positions were reported according to *Phaseolus vulgaris* v2.1 genome (DOE-JGI and USDA-NIFA^1^) positions. Raw SNPs (11,292 markers) were filtered to eliminate markers that were heterozygous or monomorphic (9,085 markers), duplicates (1,301 markers), or exhibiting extreme segregation distortion (*p*-value < 1e^–10^; 31 markers).

### Linkage and QTL Mapping

Linkage mapping was performed using MapDisto version 2.1.7 (Heffelfinger et al., 2017). Markers causing excessive map length and/or exhibiting aberrant segregation distortion patterns were excluded (five markers). A fixed-order genetic map of 439.53 cM was generated using the Kosambi function with the remaining 870 markers. Markers were grouped by chromosome with marker order reflecting physical positions. The Ripple function was used to confirm marker order.

Quantitative trait loci mapping was performed using QTL Cartographer version 2.5 (Wang et al., 2012). The composite interval mapping (CIM) procedure was performed with the parameters set to 10 cM window size and 1 cM walkspeed with forward and backward regression. BLUPs were used for all traits in QTL mapping for both years combined and for sensory traits for individual years, and means were used for analyses of all other traits for individual years. The LOD thresholds for each trait in each year and across years were determined using 1,000 permutations in scanone from rQTL with the extended Haley–Knott method (*p*-value < 0.05) (Broman et al., 2003; Feenstra et al., 2006). QTL regions were defined including all significant markers for each QTL peak. LOD information by position is available for each trait in the Supplementary Material. The constructed linkage maps with QTL overlaid were visualized using Mapchart 2.32 (Voorrips, 2002). Each QTL was named according to the guidelines for common bean QTL nomenclature (Miklas and Porch, 2010).

## Results

### Cooking Time Evaluation

Genotype and year significantly affected water uptake, and cooking time (*p*-value < 0.05) and genotype by year significantly affected cooking time (*p*-value < 0.05) (Table 1 and Supplementary Table 1). The parental lines, Ervilha and PI527538 had water uptakes of 109.3 and 98.8% and cooking times of 21.0 and 29.7 min, respectively averaged across both years.

**TABLE 1:** Parental phenotypes, means with standard error, ranges, and broad-sense heritability (H^2^) estimates for the RILs for both years combined with ANOVA *p*-values for genotype, year, and genotype by year indicated.

Trait	Ervilha	PI527538	Mean^a^	Range	H^2^	Genotype	Year	Genotype × Year
Water uptake (%)	109.3^a^ ± 3.5	98.8^a^ ± 1.2	101.64 ± 0.3	69.2–117.4	0.25	< 0.0001	<0.0001	NS
Cooking time (min)	21.0^b^ ± 1.5	29.7^a^ ± 2.4	25.25 ± 0.2	19.1–33.9	0.68	< 0.0001	<0.0001	0.0054
Total flavor intensity (0–5)	3.1^b^ ± 0.1	3.2^a^ ± 0.1	3.28 ± 0.0	2.2–4.1	0.27	< 0.0001	NS	NS
Beany intensity (0–5)	2.2^b^ ± 0.2	3.3^a^ ± 0.1	2.87 ± 0.0	1.5–3.9	0.33	< 0.0001	NS	NS
Vegetative intensity (0–5)	2.7^a^ ± 0.1	2.5^b^ ± 0.1	2.59 ± 0.0	1.7–3.4	0.05	0.0020	NS	NS
Earthy intensity (0–5)	2.0^b^ ± 0.0	2.2^a^ ± 0.0	2.23 ± 0.0	1.5–3.1	0.06	0.0010	NS	NS
Starchy intensity (0–5)	3.6^a^ ± 0.0	3.0^b^ ± 0.1	3.17 ± 0.0	2.5–3.9	0.13	< 0.0001	NS	NS
Sweet intensity (0–5)	2.3^a^ ± 0.1	1.8^b^ ± 0.1	2.05 ± 0.0	1.3–3.2	0.19	< 0.0001	NS	NS
Bitter intensity (0–5)	1.4^b^ ± 0.0	1.9^a^ ± 0.1	1.7 ± 0.0	1.1–2.3	0.14	< 0.0001	NS	NS
Seed-coat perception (0–5)	2.8^b^ ± 0.1	3.4^a^ ± 0.0	3.05 ± 0.0	2.4–3.9	0.21	< 0.0001	NS	NS
Cotyledon texture (0–5)	2.4^a^ ± 0.1	2.0^b^ ± 0.1	2.29 ± 0.0	1.4–3.0	0.06	< 0.0001	NS	< 0.0001
L*	64.8^a^ ± 0.2	54.1^b^ ± 1.8	58.8 ± 0.3	40.3–67.3	0.86	< 0.0001	<0.0001	.
a*	−0.7^b^ ± 0.6	3.5^a^ ± 0.2	1.4 ± 0.1	−3.2 to 5.9	0.86	< 0.0001	<0.0001	.
b*	22.3^a^ ± 0.9	14.6^b^ ± 2.3	20.2 ± 0.3	8.5–34.4	0.78	< 0.0001	<0.0001	.
Seed-coat postharvest non-darkening (0 = non-darkening; 1 = darkening)	0^b^ ± 0	1^a^ ± 0	0.5 ± 0.0	0–1	1.00	< 0.0001	.	.

*^*a*^Mean separation is indicated by letter superscript. Least squares estimates are presented for sensory attribute intensities instead of means.*

*NS indicates non-significant *p*-values at α = 0.05.*

**FIGURE 2:**
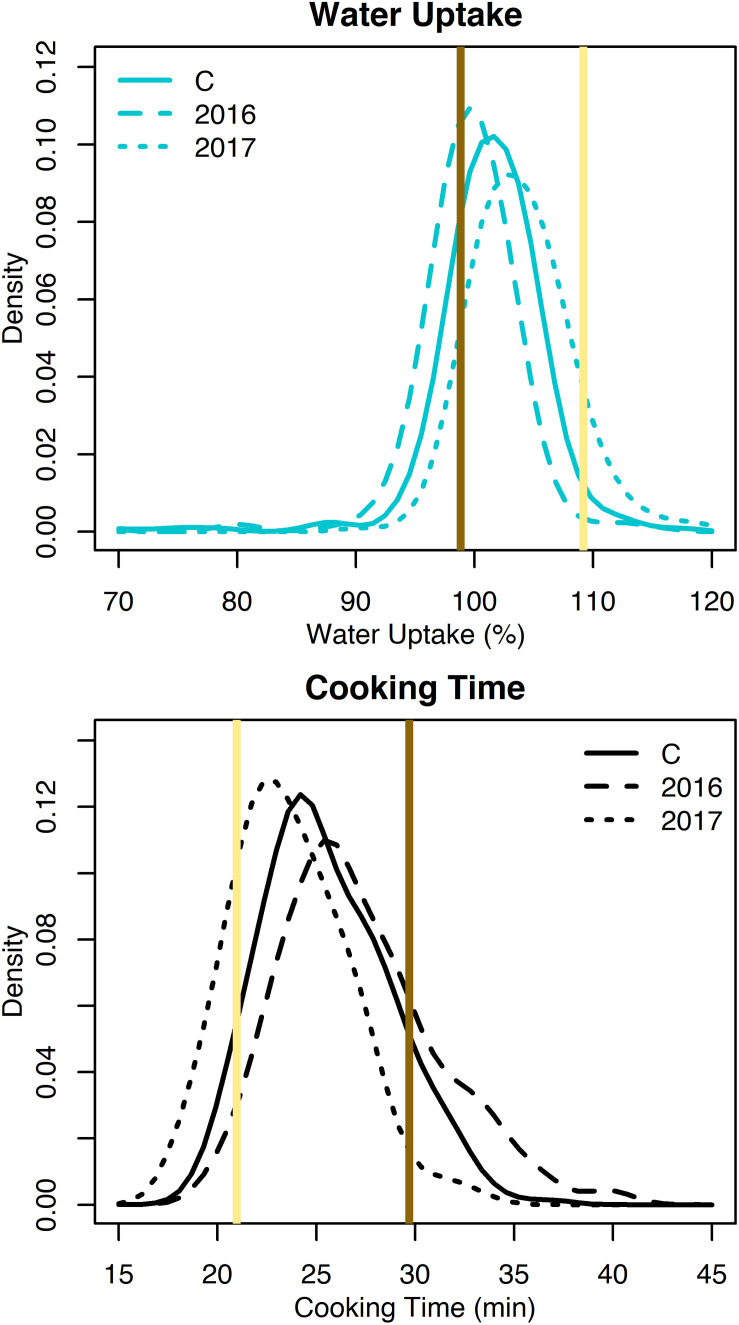
Density plots of water uptake and cooking time for the RILs from 2016, 2017, and both years combined (C). Means for Ervilha and PI527538 from both years combined are indicated in yellow and brown, respectively.

Water uptake and cooking time for the RILs exhibited approximately normal distributions (Supplementary Table 2 and Figure 2). Averaged across both years, water uptake ranged 69.2–117.4%, and cooking time ranged 19.1–33.9 min (Table 1). Broad-sense heritability for cooking time was 0.68 and water uptake was 0.25 (Table 1).

### Sensory Evaluation

Genotype significantly affected all sensory attributes (*p*-value < 0.05) (Table 1). Year did not significantly affect any sensory attributes, and genotype by year only significantly affected cotyledon texture (*p*-value < 0.05). Rep effects were insignificant for all sensory attributes, which indicates panelists were consistent across reps, although significant panelist and session effects were observed (Supplementary Table 3). For the parents Ervilha and PI527538, respectively with least squares estimates averaged across both years, the total flavor intensities were 3.1 and 3.2; beany intensities were 2.2 and 3.3; vegetative intensities were 2.7 and 2.5; earthy intensities were 2.0 and 2.2; starchy intensities were 3.6 and 3.0; sweet intensities were 2.3 and 1.8; bitter intensities were 1.4 and 1.9; seed-coat perceptions were 2.8 and 3.4; and cotyledon textures were 2.4 and 2.0 (Table 1).

**FIGURE 3:**
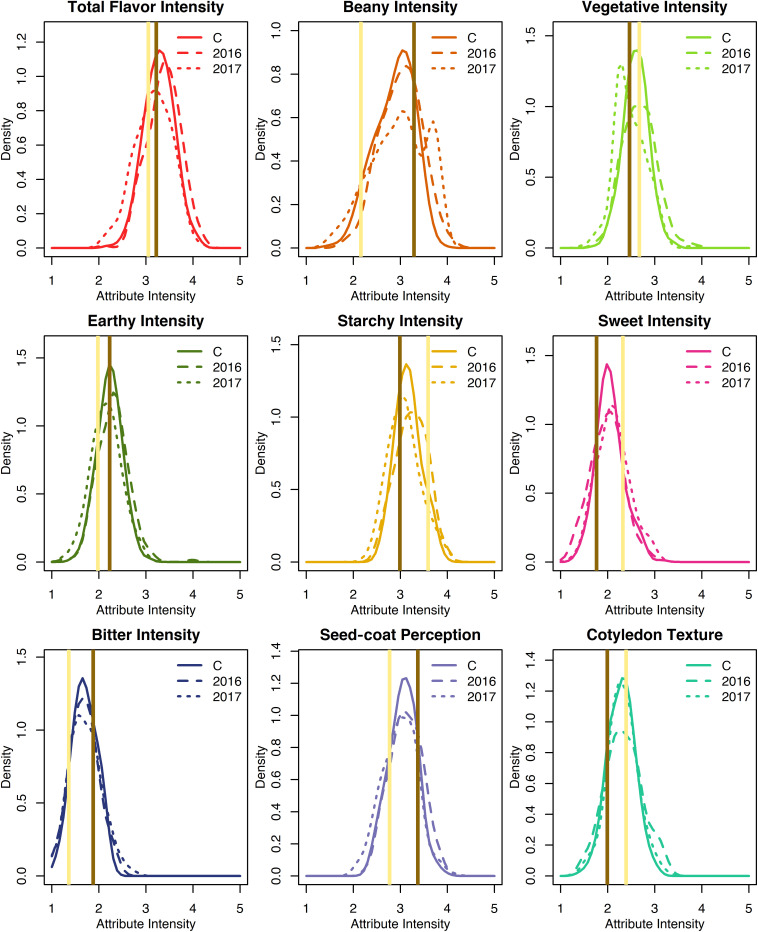
Density plots of least squares estimates of sensory attribute intensities for the RILs from 2016, 2017, and both years combined (C). Attribute intensities for Ervilha and PI527538 from both years combined are indicated in yellow and brown, respectively.

Least squares estimates for all sensory attribute intensities varied minimally across years and exhibited approximately normal distributions (Supplementary Table 2 and Figure 3). Across both years, least squares estimates ranged 2.2–4.1 for total flavor intensity, 1.5–3.9 for beany intensity, 1.7–3.4 for vegetative intensity, 1.5–3.1 for earthy intensity, 2.5–3.9 for starchy intensity, 1.3–3.2 for sweet intensity, 1.1–2.3 for bitter intensity, 2.4–3.9 for seed-coat perception, and 1.4–3.0 for cotyledon texture (Table 1). While panelists were able to differentiate among genotypes using 5-point scales, sensory attribute ranges did not exceed 2.4, suggesting panelists did not make full use of the scales. This could reflect the limited differences in sensory attribute intensities observed between the parents.

Broad-sense heritability for sensory attribute intensities were low, ranging from 0.05 to 0.33 (Table 1). Beany intensity and total flavor intensity exhibited the highest broad-sense heritability (0.33 and 0.27), while vegetative intensity, earthy intensity, and cotyledon texture exhibited the lowest (0.05, 0.06, and 0.06).

### Color and Seed-Coat Postharvest Darkening

Genotype significantly affected L^∗^, a^∗^, b^∗^, and seed-coat postharvest non-darkening (*p*-value < 0.05) (Table 1). Year significantly affected L^∗^, a^∗^, and b^∗^ (*p*-value < 0.05). For the parents Ervilha and PI527538, respectively averaged across both years, L^∗^ values were 64.8 and 54.1; a^∗^ values were −0.7 and 3.5; b^∗^ values were 22.3 and 14.6; and seed-coat postharvest non-darkening values were 0 (non-darkening) and 1 (darkening).

**FIGURE 4:**
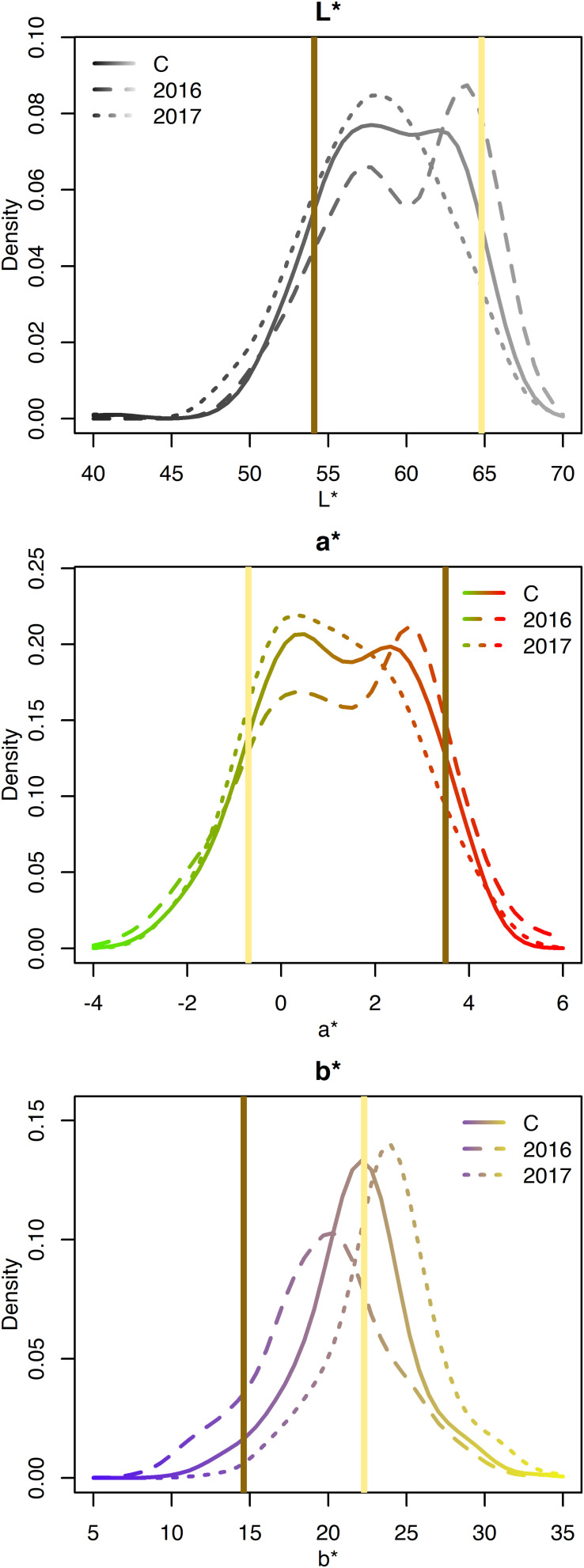
Density plots of CIELAB values for the RILs from 2016, 2017, and both years combined (C). Attribute intensities for Ervilha and PI527538 from both years combined are indicated in yellow and brown, respectively.

The L^∗^, a^∗^, and b^∗^ for the RILs varied minimally across years and exhibited approximately normal distributions (Supplementary Table 2 and Figure 4). Averaged across both years, L^∗^ ranged from 40.3–67.3; a^∗^ ranged from −3.2 to 5.9; and b^∗^ ranged from 8.5 to 34.4 (Table 1). Seed-coat postharvest darkening was only determined for seeds from 1 year (2017), and progeny exhibiting both non-darkening and darkening were observed. Broad-sense heritability was high for L^∗^ (0.86), a^∗^ (0.86), b^∗^ (0.78), and seed-coat postharvest non-darkening (1.00).

### Seed Yield and Seed Weight

Genotype, year, and genotype by year significantly affected seed weight and seed yield (*p*-value < 0.05) (Supplementary Table 1). For the parents Ervilha and PI527538, respectively averaged across both years, the seed weights were 52.8 and 48.0 g per 100 seeds. Seed yield data for Ervilha is not available for 2016 (Supplementary Table 2), and fewer seeds were planted per plot in 2016, making averages across years misleading. In 2017, the seed yields for Ervilha and PI527538, respectively, were 1,731.4 and 2,384.4 kg/ha.

The seed weight for the RILs exhibited approximately normal distributions (Supplementary Table 2 and Supplementary Figure 1). Seed yield for the RILs varied substantially across years due to reduced seeds planted per plot in 2016 but exhibited approximately normal distributions. Averaged across both years, seed weight ranged 39.1–68.4 g per 100 seeds and seed yield ranged 751.0–3,283.9 kg per ha (Supplementary Table 2).

Broad-sense heritability for seed weight (0.89) was high and for seed yield was moderate (0.57) (Supplementary Table 1).

### Principal Component Analysis

**FIGURE 5:**
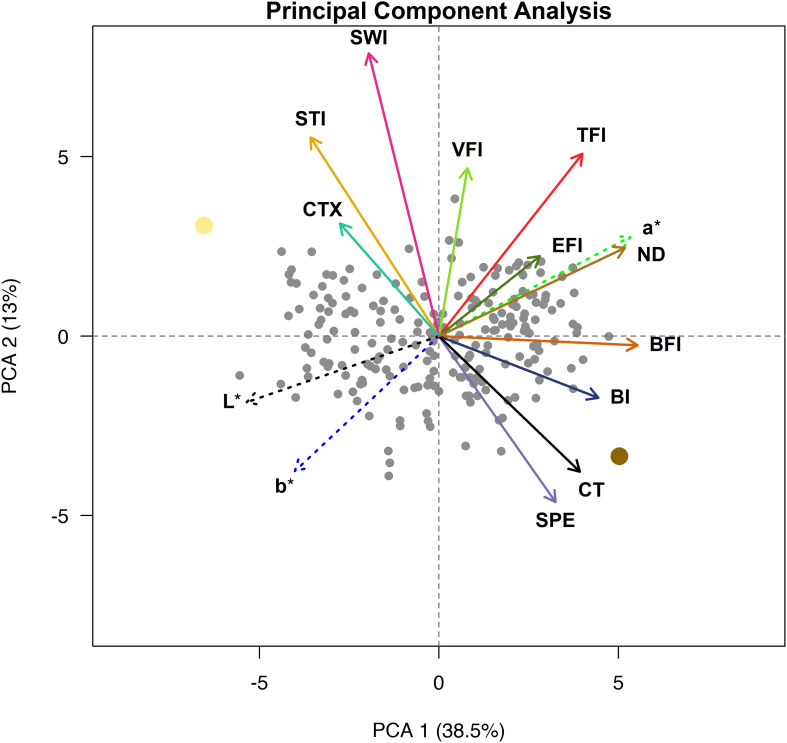
Principal component analysis biplot with loadings for cooking time (CT), total flavor intensity (TFI), beany intensity (BFI), vegetative intensity (VFI), earthy intensity (EFI), starchy intensity (STI), sweet intensity (SWI), bitter intensity (BI), seed-coat perception (SPE), cotyledon texture (CTX), L*, a*, and b*. Ervilha and PI527538 are indicated in yellow and brown, respectively.

A PCA for the seed quality trait relationship was conducted and the first two principal components (PCs) explained approximately 52% of the variance (Figure 5). The first PC separates the genotypes approximately by beany, earthy, and bitter intensities as well as L^∗^, a^∗^, b^∗^, and seed-coat postharvest non-darkening and represents over a third of the variation (38.5%). The second PC separates the genotypes approximately by cooking time; total flavor, vegetative, starchy, and sweet intensities; and cotyledon texture and seed-coat perception. The second PC represents over an eighth of the variance (13.0%). The remaining PCs accounted for 11.1, 7.4, 6.0, 5.4, 4.2, 3.2, 2.9, 2.5, 2.2, 1.6, 1.1, and 0.9% of the variance, respectively (data not shown).

The PCA biplot highlights distinct groupings of traits that tend to be observed together. Loadings that group together highlight strong positive relationships within each group, and groups of loadings opposite of each other highlight strong negative relationships between groups. Loadings for starchy intensity, sweet intensity, and cotyledon texture are positioned close to each other and opposite cooking time and seed-coat perception. Loadings for beany intensity and bitter intensity also group together and are somewhat opposite starchy intensity, sweet intensity, and cotyledon texture. The loadings for total flavor intensity earthy intensity, a^∗^, and seed-coat postharvest non-darkening group together, opposite of loadings for L^∗^ and b^∗^. The loading for vegetative intensity does not appear to group with or opposite of other loadings but lies in between loadings for total flavor intensity and sweet intensity. The genotypes are spread across the biplot, with Ervilha and PI527538 positioned opposite each other.

### QTL Mapping

**FIGURE 6:**
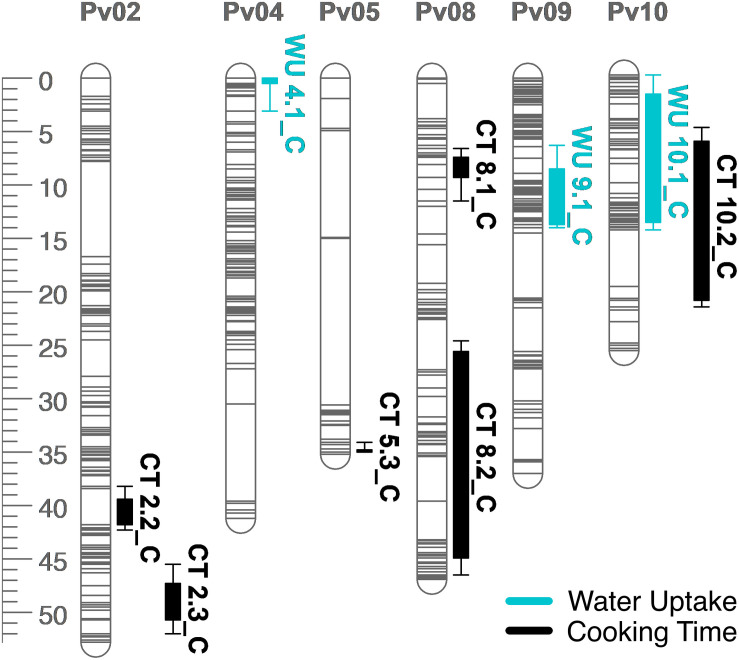
QTL map for water uptake (WU) and cooking time (CT) in the RIL population. Size is in cM. All QTL were detected in both years combined.

**FIGURE 7:**
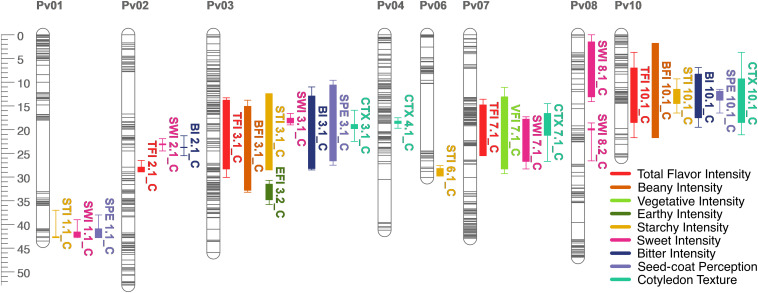
QTL map for total flavor intensity (TFI), beany intensity (BFI), vegetative intensity (VFI), earthy intensity (EFI), starchy intensity (STI), sweet intensity (SWI), bitter intensity (BI), seed-coat perception (SPE), and cotyledon texture (CTX) in the RIL population. Size is in cM. All QTL were detected in both years combined.

**FIGURE 8:**
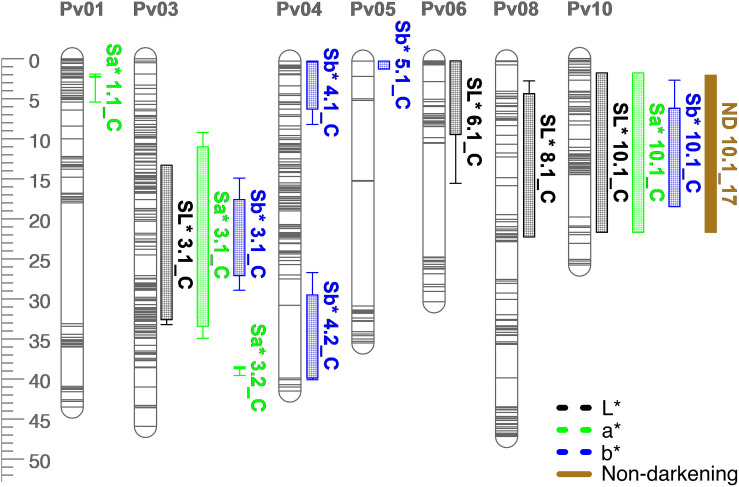
QTL map for L*, a*, b*, and seed-coat postharvest non-darkening (ND) in the RIL population. Size is in cM. All QTL were detected in both years combined.

A linkage map was developed with 870 SNPs spread across eleven chromosomes for a total map length of 439.53 cM with a marker density of one SNP per 0.51 cM (Table 2). Significant QTL were identified using BLUPs from both years combined for water uptake, cooking time, total flavor intensity, beany intensity, vegetative intensity, earthy intensity, starchy intensity, sweet intensity, bitter intensity, seed-coat perception, cotyledon texture, L^∗^, a^∗^, b^∗^, seed-coat postharvest non-darkening, seed weight, and seed yield (Tables 3–5, Figures 6–8, and Supplementary Figure 2). Additional QTL were also identified in individual years for these traits (Supplementary Tables 4–7).

**TABLE 2:** Linkage map information for the 242 RILs.

Linkage group	Number of markers	Size		Marker density	
			
		cM	Mb	cM/marker	Mb/marker
Pv01	72	43.50	51.28	0.60	0.71
Pv02	117	52.82	48.64	0.45	0.42
Pv03	138	45.86	52.29	0.33	0.38
Pv04	97	41.20	47.96	0.42	0.49
Pv05	24	35.22	40.46	1.47	1.69
Pv06	40	30.05	24.56	0.75	0.61
Pv07	120	42.89	39.88	0.36	0.33
Pv08	78	46.88	62.74	0.60	0.80
Pv09	100	36.99	36.26	0.37	0.36
Pv10	60	25.79	43.98	0.43	0.73
Pv11	24	38.32	52.41	1.60	2.18
Total	870	439.53	500.46	0.51	0.58

**TABLE 3:** Quantitative trait loci identified in the RIL population (*N* = 242) using BLUPs from samples grown in Entrican, MI in 2016 and 2017 for water uptake and cooking time.

Trait	QTL name	LG	Pos (bp)	Pos (cM)	LOD	*R*^2^ (%)	a^a^	Physical interval^b^ (Mb)	Map interval^c^ (cM)	Sig^d^
Water uptake	WU4.1	Pv04	53,507	0.01	2.8	4.1	−	0.05–0.24	0.01–0.50	**
	WU9.1	Pv09	13,800,234	10.76	3.7	5.7	+	12.60–16.11	8.46–13.66	**
	WU10.1	Pv10	41,060,322	11.99	5.5	9.2	+	3.45–42.26	1.83–13.77	**
Cooking time	CT2.2	Pv02	44,116,993	40.44	3.6	5.6	−	42.75–44.12	39.44–41.82	**
	CT2.3	Pv02	47,982,820	50.60	3.6	4.5	−	46.45–48.35	47.31–50.69	**
	CT5.3	Pv05	40,651,928	35.04	3.7	4.7	−	40.52–40.68	34.76–35.04	**
	CT8.1	Pv08	1835,444	9.12	2.8	3.6	−	1.26–2.17	7.35–9.29	*
	CT8.2	Pv08	60,515,678	36.45	7.5	10.2	+	53.03–62.50	25.56–44.90	**
	CT10.2	Pv10	41,060,322	11.99	15.6	22.3	−	37.83–43.84	6.17–21.13	**

*Linkage group (LG), year, peak position (Pos), logarithm of odds (LOD), *R*^2^, QTL effect (a), physical interval, map interval, and significance of the QTL are indicated.*

*The largest LOD and *R*^2^ within the QTL are reported.*

*^*a*^+ and − indicate positive and negative effects on the mean as conferred by alleles from Ervilha in the QTL region.*

*^*b*^Physical positions of the nearest markers upstream and downstream of the map interval.*

*^*c*^Region where LOD scores are significant at the indicated significance level.*

^*d*^Significance at α = 0.1 and α = 0.05 are indicated by * and **, respectively, based on 1,000 permutations.

**TABLE 4:** Quantitative trait loci identified in the RIL population (*N* = 242) using BLUPs from samples grown in Entrican, MI in 2016 and 2017 for sensory attributes. Linkage group (LG), year, peak position (Pos), logarithm of odds (LOD), *R*^2^, QTL effect (a), physical interval, map interval, and significance of the QTL are indicated.

Trait	QTL Name	LG	Pos (bp)	Pos (cM)	LOD	*R*^2^ (%)	a^a^	Physical interval^b^ (Mb)	Map interval^c^ (cM)	Sig^d^
Total flavor intensity	TFI2.1	Pv02	31,802,612	27.92	3.1	3.9	−	31.80–33.08	27.92–28.90	**
	TFI3.1	Pv03	41,406,149	27.09	5.0	6.1	−	4.16–43.22	13.80–28.25	**
	TFI7.1	Pv07	6,719,315	17.15	8.1	10.9	+	3.79–34.27	14.77–25.45	**
	TFI10.1	Pv10	41,060,322	11.99	13.2	18.4	−	38.84–43.47	7.03–18.53	**
Beany intensity	BFI3.1	Pv03	32,070,949	18.90	11.0	13.1	−	5.95–48.71	15.12–32.83	**
	BFI10.1	Pv10	41,060,322	11.99	18.8	25.1	−	3.45–43.85	1.83–21.71	**
Vegetative intensity	VFI7.1	Pv07	6,719,315	17.15	5.8	9.3	+	1.62–36.08	13.11–28.30	**
Earthy intensity	EFI3.2	Pv03	48,019,631	32.64	3.8	5.9	−	46.42–49.58	31.48–34.77	**
Starchy intensity	STI1.1	Pv01	51,140,837	42.57	3.4	4.6	+	50.95–51.19	42.57–42.65	**
	STI3.1	Pv03	30,678,469	18.71	8.9	12.6	+	3.76–43.47	12.37–28.44	**
	STI6.1	Pv06	30,971,026	29.77	3.7	5.1	+	30.66–30.97	28.19–29.77	**
	STI10.1	Pv10	42,224,711	13.77	5.1	6.9	+	40.90–43.47	11.51–14.53	**
Sweet intensity	SWI1.1	Pv01	50,948,757	41.57	2.7	3.8	+	50.95–51.14	41.57–42.57	*
	SWI2.1	Pv02	25,666,553	22.99	3.0	4.1	+	25.67–27.31	22.99–23.18	**
	SWI3.1	Pv03	29,623,010	17.61	3.8	5.4	+	29.62–30.68	17.61–18.61	**
	SWI7.1	Pv07	29,986,177	21.23	6.2	9.9	+	8.02–34.78	17.82–26.72	**
	SWI8.1	Pv08	2,171,710	10.37	5.1	5.4	+	0.13–4.00	1.49–13.05	**
	SWI8.2	Pv08	6,802,573	19.82	3.0	3.9	+	6.80–7.97	19.82–20.10	**
Bitter intensity	BI2.1	Pv02	27,306,217	23.66	3.1	5.0	−	27.31–28.36	23.66–23.75	**
	BI3.1	Pv03	32,070,949	18.90	7.4	10.6	−	3.84–42.96	12.85–28.15	**
	BI10.1	Pv10	40,959,742	11.90	5.4	8.7	−	39.72–43.47	8.30–17.53	**
Seed-coat perception	SPE1.1	Pv01	51,140,837	42.57	4.1	5.7	−	50.83–51.33	40.92–42.84	**
	SPE3.1	Pv03	32,070,949	18.90	13.3	20.1	−	3.00–41.41	10.59–26.55	**
	SPE10.1	Pv10	40,959,742	11.90	2.8	4.0	−	40.96–42.26	11.90–13.77	*
Cotyledon texture	CTX3.1	Pv03	32,803,416	19.00	2.8	4.1	+	32.07–33.78	18.90–19.76	*
	CTX4.1	Pv04	7,368,228	18.23	3.1	4.7	+	7.37–9.45	18.23–18.71	**
	CTX7.1	Pv07	8,690,008	18.10	4.1	6.0	+	5.45–29.99	16.58–21.23	**
	CTX10.1	Pv10	42,451,057	14.34	6.4	9.6	+	39.72–43.47	9.30–18.53	**

*The largest LOD and *R*^2^ within the QTL are reported.*

*^*a*^+ and − indicate positive and negative effects on the mean as conferred by alleles from Ervilha in the QTL region.*

*^*b*^Physical positions of the nearest markers upstream and downstream of the map interval.*

*^*c*^Region where LOD scores are significant at the indicated significance level.*

^*d*^Significance at α = 0.1 and α = 0.05 are indicated by * and **, respectively, based on 1,000 permutations.

**TABLE 5:** Quantitative trait loci identified in the RIL population (*N* = 242) using BLUPs from samples grown in Entrican, MI in 2017 for color and seed-coat postharvest non-darkening.

Trait	QTL name	LG	Pos (bp)	Pos (cM)	LOD	*R*^2^ (%)	a^a^	Physical interval^b^ (Mb)	Map interval^c^ (cM)	Sig^d^
L*	SL*3.1	Pv03	39,674,302	25.6	7.8	4.5	+	3.91–48.14	13.32–32.64	**
	SL*6.1	Pv06	17,329,576	4.9	8.2	4.5	+	6.42–20.51	0.01–9.19	**
	SL*8.1	Pv08	3,007,875	12.1	10.4	5.8	+	0.63–24.47	4.11–22.00	**
	SL*10.1	Pv10	40,959,742	11.9	60.0	55.6	+	3.45–43.85	1.83–21.71	**
a*	Sa*1.1	Pv01	1,333,499	2.2	3.2	2.4	+	1.33–1.42	2.21–2.31	**
	Sa*3.1	Pv03	43,470,352	28.5	6.0	4.4	−	3.16–48.92	10.96–33.41	**
	Sa*3.2	Pv03	51,330,379	38.5	3.3	2.5	−	51.33–52.15	38.48–38.58	**
	Sa*10.1	Pv10	40,959,742	11.9	44.9	51.9	−	3.45–43.85	1.83–21.71	**
b*	Sb*3.1	Pv03	39,287,195	23.8	4.7	5.9	+	29.62–41.84	17.61–27.09	**
	Sb*4.1	Pv04	421,790	0.8	3.4	4.2	+	0.13–2.06	0.50–6.02	**
	Sb*4.2	Pv04	45,728,204	34.6	4.7	8.3	+	45.04–47.65	29.16–39.6	**
	Sb*5.1	Pv05	219,601	0.0	3.0	3.7	−	0.22–0.52	0.01–1.01	**
	Sb*10.1	Pv10	41,510,878	12.7	12.7	17.2	+	37.83–43.47	6.17–18.53	**
Seed-coat postharvest non-darkening^*e*^	ND10.1^WP,YY^	Pv10	43,465,901	18.5	81.2^f^	87.5	−	3.98–43.85	2.11–21.71	**

*Linkage group (LG), year, peak position (Pos), logarithm of odds (LOD), *R*^2^, QTL effect (a), physical interval, map interval, and significance of the QTL are indicated.*

*The largest LOD and *R*^2^ within the QTL are reported.*

*^*a*^+ and − indicate positive and negative effects on the mean as conferred by alleles from Ervilha in the QTL region.*

*^*b*^Physical positions of the nearest markers upstream and downstream of the map interval.*

*^*c*^Region where LOD scores are significant at the indicated significance level.*

^*d*^Significance at α = 0.1 and α = 0.05 are indicated by * and **, respectively, based on 1,000 permutations. ^*e*^Seed-coat postharvest non-darkening was only evaluated on samples grown in 2017. ^*f*^Many LODs in the map interval for ND10.1 were not able to be reported by QTL Cartographer so the highest reported LOD is indicated.

**FIGURE 9:**
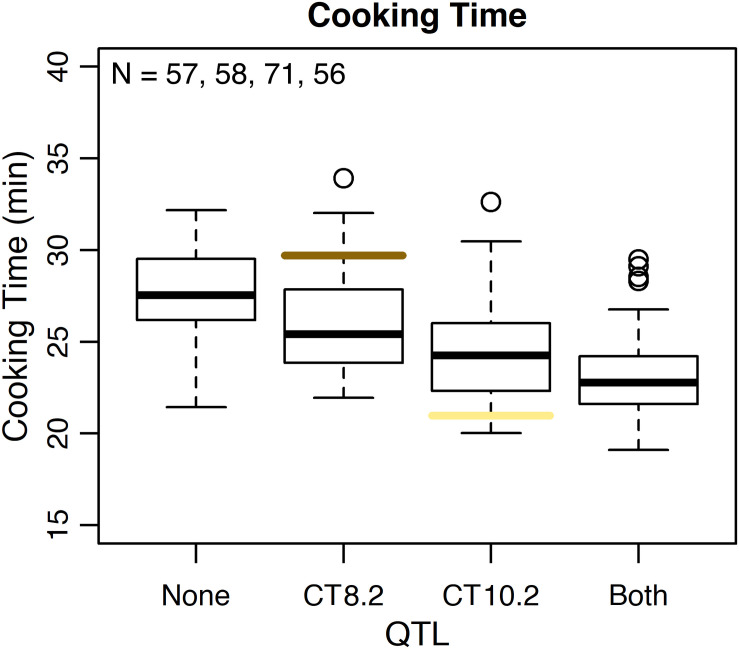
Phenotypic effect of CT8.2 and CT10.2 in the RIL population based on the peak SNP alleles and average cooking time across both years. Cooking times for RILs with no fast-cooking alleles (none), the fast-cooking CT8.2 allele (CT8.2), the fast-cooking CT10.2 allele (CT10.2), and both fast-cooking alleles (both) are displayed in boxplots. Means for Ervilha and PI527538 from both years combined are indicated in yellow and brown, respectively.

Several QTL were identified for water uptake and cooking time in both years combined. For water uptake, three QTL were identified: WU4.1, WU9.1, and WU10.1 (Table 3 and Figure 6). The total proportion of variance explained by the three QTL was 19.0%. For cooking time, six QTL were identified: CT2.2, CT2.3, CT5.3, CT8.1, CT8.2, and CT10.2 (Table 3 and Figure 6). The total proportion of variance explained by the six QTL was 50.9%. CT8.2 and CT10.2 were the most significant cooking time QTL identified. RILs with both fast-cooking alleles for these QTL cooked 5 min faster on average than RILs without fast-cooking alleles (Figure 9).

Many QTL were identified across all sensory characteristics in both years combined. For total flavor intensity, four QTL were identified: TFI2.1, TFI3.1, TFI7.1, and TFI10.1 (Table 4 and Figure 6). The proportion of variance explained by the four QTL was 39.3%. For beany intensity, two QTL were identified: BFI3.1 and BFI10.1 (Table 4 and Figure 7). The proportion of the variance explained by the two QTL was 38.2%. For vegetative intensity, one QTL was identified: VFI7.1 (Table 4 and Figure 7). The proportion of variance explained by VFI7.1 was 9.3%. For earthy intensity, one QTL was identified: EFI3.2 (Table 4 and Figure 7). The proportion of variance explained by EFI3.2 was 5.9%. For starchy intensity, four QTL were identified: STI1.1, STI3.1, STI6.1, and STI10.1 (Table 4 and Figure 7). The proportion of variance explained by the four QTL was 29.2%. For sweet intensity, six QTL were identified: SWI1.1, SWI2.1, SWI3.1, SWI7.1, SWI8.1, and SWI8.2 (Table 4 and Figure 7). The proportion of variance explained by the six QTL was 32.5%. For bitter intensity, three QTL were identified: BI2.1, BI3.1, and BI10.1 (Table 4 and Figure 7). The proportion of variance explained by the three QTL was 24.3%. For seed-coat perception, three QTL were identified: SPE1.1, SPE3.1, and SPE10.1 (Table 4 and Figure 7). The proportion of variance explained by the three QTL was 29.8%. For cotyledon texture, four QTL were identified: CTX3.1, CTX4.1, CTX7.1, and CTX10.1 (Table 4 and Figure 7). The proportion of variance explained by the four QTL was 24.4%.

Many QTL were identified for color traits across both years combined. For L^∗^, four QTL were identified: SL^∗^3.1, SL^∗^6.1, SL^∗^8.1, and SL^∗^10.1 (Table 5 and Figure 8). The total proportion of variance explained by four QTL was 70.4%. For a^∗^, four QTL were identified: Sa^∗^1.1, Sa^∗^3.1, Sa^∗^3.2, and Sa^∗^10.1 (Table 5 and Figure 8). The total proportion of variance explained by the four QTL was 61.2%. For b^∗^, five QTL were identified: Sb^∗^3.1, Sb^∗^4.1, Sb^∗^4.2, Sb^∗^5.1, and Sb^∗^10.1 (Table 5 and Figure 8). The total proportion of variance explained by the five QTL was 39.3%. For seed-coat postharvest non-darkening, one QTL was identified: ND.10.1 (Table 5 and Figure 8). Seed-coat postharvest non-darkening was only evaluated for 2017 seeds. The proportion of variance explained by ND10.1 was 87.5%, and Ervilha contributed an allele conferring a negative effect, reflecting its lack of darkening over time (Tables 1, 5).

While seed weight and seed yield were not central to this study, several QTL were identified for these traits as well. Additional information is available in the supplementary material (Supplementary Figure 2 and Supplementary Table 7).

Several QTL co-localized on Pv01, Pv03, Pv04, Pv07, Pv08, and Pv10. On Pv01, QTL for starchy intensity (STI1.1), sweet intensity (SWI1.1), and seed-coat perception (SPE1.1) co-localized. Alleles from Ervilha conferred positive effects for STI1.1 and SWI1.1 and a negative effect for SPE1.1. On Pv03, QTL for total flavor intensity (TFI3.1), beany intensity (BFI3.1), earthy intensity (EFI3.1 and EFI3.2), starchy intensity (STI3.1), sweet intensity (SWI3.1), bitter intensity (BI3.1), seed-coat perception (SPE3.1), cotyledon texture (CTX3.1), L^∗^ (SL^∗^3.1), a^∗^ (Sa^∗^3.1), and b^∗^ (Sb^∗^3.1) co-localized. Alleles from Ervilha conferred positive effects for STI3.1, SWI3.1, CTX3.1, SL^∗^3.1, and Sb^∗^3.1 and negative effects for TFI3.1, BFI3.1, EFI3.1, EFI3.2, BI3.1, SPE3.1, and Sa^∗^3.1. On Pv04, QTL for water uptake (WU4.1 and WU4.2) and b^∗^ (Sb^∗^4.1 and Sb^∗^4.2) co-localized. Alleles from Ervilha conferred positive effects for WU4.2, Sb^∗^4.1, and Sb^∗^4.2 and a negative effect for WU4.1. On Pv07, QTL for total flavor intensity (TFI7.1), vegetative intensity (VFI7.1), sweet intensity (SWI7.1), and cotyledon texture (CTX7.1 and CTX7.2) co-localized. Alleles from Ervilha conferred positive effects for TFI7.1, VFI7.1, SWI7.1, CTX7.1, and CTX7.2. On Pv08, QTL for cooking time (CT8.1), starchy intensity (STI8.1), sweet intensity (SWI8.1 and SWI8.2), and L^∗^ (SL^∗^8.1) co-localized. Alleles from Ervilha conferred positive effects for STI8.1, SWI8.1, SWI8.2, and SL^∗^8.1 and a negative effect for CT8.1. On Pv10, QTL for water uptake (WU10.1), cooking time (CT10.2), total flavor intensity (TFI10.1), beany intensity (BFI10.1), starchy intensity (STI10.1), bitter intensity (BI10.1), seed-coat perception (SPE10.1), cotyledon texture (CTX10.1), L^∗^ (SL^∗^10.1), a^∗^ (Sa^∗^10.1), b^∗^ (Sb^∗^10.1), and seed-coat postharvest non-darkening (ND10.1) co-localized. Alleles from Ervilha conferred positive effects for WU10.1, STI10.1, CTX10.1, SL^∗^10.1, and Sb^∗^10.1 and negative effects for CT10.2, TFI10.1, BFI10.1, BI10.1, SPE10.1, Sa^∗^10.1, and ND10.1.

## Discussion

The broad-sense heritability for cooking time was moderately high in this study, as was the case for previous reports looking at both broad-sense and narrow-sense heritability (Elia et al., 1997; Jacinto-Hernandez et al., 2003; Cichy et al., 2019; Bassett et al., 2020b). This supports the idea that marker-assisted selection for fast cooking time may be feasible with few molecular markers. Using marker-assisted selection as opposed to phenotyping could save breeding programs time and prevent the need to purchase specialized machinery specific for the evaluation of cooking time. It could also allow for early generation screening that would otherwise not be feasible due to limited seed and the large number of lines to be evaluated for cooking time.

Differences in sensory attribute intensities among genotypes were successfully detected, allowing the relationship among attributes in this population to be determined and for significant QTL to be identified for the evaluated sensory attributes. While significant panelist and session effects were identified (Supplementary Table 2), QDA does not rely on consensus among panelists, and these effects can be accounted for by using least squares estimates and BLUPs where appropriate. Although broad-sense heritability for sensory attributes tended to be low to very low, it is clear that genotype is important for flavor and texture. In the context of a breeding program, heritability can be improved by screening fewer lines with greater replication to better account for panelist and session effects while managing limited seed and personnel resources. As has been previously noted, panelists tend not to use the full range of the rating scales, which prevents detection of small differences between samples (Bassett et al., 2020b). In the case of this population, it is unlikely that this RIL population exhibited a full range of sensory attribute intensities, especially for traits with limited differences in the parents, so incomplete use of the scales likely reflects a lack of extreme differences among genotypes. However, increasing the size of the scales or using line scales that allow for continuous ratings may better reflect the diversity of attribute intensities exhibited in a population in future studies, which might return higher heritability for sensory traits. Year and genotype by year effects were not significant for sensory traits, apart from cotyledon texture, which had a significant genotype by year effect. This is encouraging because location of production and crop management practices have previously been identified as factors affecting sensory quality (Mkanda et al., 2007; Ferreira et al., 2012). This indicates that flavor and texture traits do not change across years in the same production environment, which is useful for meeting expectations of consistency for consumers and for product developers, who need consistent ingredients over time for their products to be successful.

There did not appear to be distinct groupings of genotypes based on cooking time and attribute intensity in the PCA biplot, indicating that there was a general mixing of these traits in the progeny (Figure 4). This suggests that extensive efforts at breaking linkages among traits are not needed to combine desired traits and achieve a target cooking time and sensory profile. Developing new yellow bean varieties with both fast cooking time and desirable flavor and texture would address two major factors influencing consumer purchasing decisions regarding dry beans and provide novelty for the many consumers unfamiliar with the yellow seed type (Leterme and Carmenza Muñoz, 2002; Eihusen and Albrecht, 2007; Winham et al., 2019).

Many QTL were identified in this study, with those for cooking time and sensory attribute intensities of particular interest. Two cooking time QTL (CT2.3 and CT8.2) cover physical ranges including ss715646000 (Pv02 48,676,223 bp), ss715646002 (Pv02 48,704,298 bp), S08_60104796 (Pv08 60,104,796 bp), and S08_62659170 (Pv08 62,659,170 bp), which are significant SNPs previously identified *via* genome-wide association in the ADP (Cichy et al., 2015b; Bassett et al., 2020b). Another recent study identified cooking time QTL on Pv02, Pv05, and Pv10, but the physical positions are not proximal to cooking time QTL identified in this study (Berry et al., 2020). All cooking time QTL identified in this study were detected in both years combined and have potential for use in marker-assisted selection. Because the LOD and *R*^2^ values for CT8.2 and CT10.2 are particularly high, these two cooking time QTL are the most compelling for marker development. The genetic control of sensory attributes is a new area of research in dry beans with limited study (Bassett et al., 2020b). For total flavor intensity, TFI2.1, TFI3.1, and TFI10.1 cover physical ranges including or in close proximity to S02_34288083 (Pv02 34,288,083 bp), S03_36213088 (Pv03 36,213,088 bp), S10_42515259 (Pv10 42,515,259 bp), and S10_42798266 (Pv10 42,798,266 bp), which were identified in association with total flavor intensity for the ADP (Bassett et al., 2020b). In addition, BFI10.1 and CTX3.1 cover physical ranges including or in close proximity to S10_42475118 (Pv10 42,475,118 bp) and S03_31659572 (Pv03 31,659,572 bp), which were also identified in the ADP in association with beany intensity and cotyledon texture, respectively (Bassett et al., 2020b). Otherwise, the QTL identified for sensory attributes in this study were novel. While most QTL identified for flavor and texture were consistent across years and many exhibited high LOD and *R*^2^ values, further validation would be beneficial before use in marker-assisted selection.

Three QTL were identified for water uptake and 13 QTL for CIELAB values. Some water uptake and CIELAB QTL were proximal to QTL and genetic markers identified in previous studies (Cichy et al., 2014; Mendoza et al., 2017; Erfatpour et al., 2018; Bassett et al., 2020b). WU4.2 and WU10.1 are near SNPs for water uptake identified in the ADP (Bassett et al., 2020b). SL^∗^10.1, Sa^∗^10.1, and Sb^∗^10.1 overlapped with the *J*-locus associated with postharvest non-darkening (Erfatpour et al., 2018). SL^∗^8.1, SL^∗^10.1, and Sa^∗^10.1 overlapped with QTL for L^∗^ and a^∗^ of canned black beans identified by Bornowski et al. (2020). Two seed coat lightness QTL fall within the ranges of known *P. vulgaris* color genes. The V color gene responsible to violet (blue to black) color falls within the SL^∗^6.1 interval and the Gy color gene (greenish yellow coat color) falls within the SL^∗^8.1 interval (Myers et al., 2019).

Seed-coat postharvest darkening was detected in PI527538 and half of the RILs. Seed-coat postharvest darkening describes the tendency of some genotypes to darken in color over time due to the presence of proanthocyanidin precursors in the seed coat (Beninger et al., 2005; Chen et al., 2015). This phenomenon has been most studied in pinto and cranberry beans but can be observed in other market classes. Lighter seed coats are perceived by consumers as indications of freshness or quality, so seeds exhibiting postharvest darkening have reduced market value (Nasar-Abbas et al., 2009; Erfatpour and Pauls, 2020). The *J* locus was previously identified on Pv10, and genotypes that are homozygous recessive at *J* do not exhibit postharvest darkening (Bassett, 2007; Elsadr et al., 2011; Erfatpour et al., 2018). The QTL identified for the non-darkening trait in this study overlaps with a previously identified QTL for non-darkening located between 40.16 and 40.30 Mb on Pv10 (Supplementary Table 5; Erfatpour et al., 2018). Flavan-3-ols, which include proanthocyandidins, have been previously associated with bitterness and astringency depending on their degree of polymerization (Robichaud and Noble, 1990; Peleg et al., 1999), so seed-coat postharvest darkening may alter flavor over time. The relationship between seed-coat postharvest darkening and flavor after beans have darkened was not examined in this study, but it remains practical to select against darkening when developing new varieties to ensure greater visual appeal to consumers, which would bypass flavor changes caused by darkening altogether. A SNP-based marker has been developed to allow marker-assisted selection for this trait (Erfatpour and Pauls, 2020).

As there is still much to be understood regarding flavor and texture in dry beans, other methods for assessing these sensory traits like GC-MS and texture measurements should be explored. Volatile concentrations and texture measurements have been used successfully as proxies for flavor and texture in studies looking at genetic control of sensory traits in other crops, and these measurements can be cheaper and easier to obtain than those generated by a descriptive panel (Zhang et al., 2015; Amyotte et al., 2017; Bauchet et al., 2017; Zhao et al., 2019). Apart from beany intensity (Vara-Ubol et al., 2004; Bott and Chambers, 2006), however, the contribution of volatiles to perceived flavors in dry beans is not well understood, and texture measurements have not been well explored outside of their use in the evaluation of firmness in canned samples (Kelly and Cichy, 2012). In addition, research assessing consumer preference for flavor and texture in dry beans is needed to define breeding targets for sensory attributes. Understanding which traits are most important for consumer preference and what the expectations are for different seed types will help breeders address flavor and texture with a focused, efficient approach.

Dry beans in the United States are sold as market classes rather than variety preserved. Variation exists within market classes for consumer-valued traits like cooking time, flavor, and texture so consumers are not able to make informed purchasing decisions taking these traits into account (Cichy et al., 2015b; Bassett et al., 2020b; Berry et al., 2020). In addition, the canning industry cannot receive the benefits of reduced energy costs and higher efficiency associated with fast-cooking genotypes if slow-cooking genotypes are present in the same cans (Bassett et al., 2020a). Because yellow beans are largely unfamiliar to United States consumers, there is an opportunity to develop new yellow bean varieties that prioritize these traits so that the yellow color can serve as a marker for convenience and culinary quality to consumers and the canning industry can produce quality canned products with yellow beans while benefitting from shorter processing times. Consumers are already seeking out unique flavors, textures, seed patterns, and colors from heirloom beans (Bullard, 2016), but heirlooms are not suited to modern farming practices, which makes them more expensive and less widely available than more familiar market classes. Yellow beans, the Manteca market class in particular, could serve this consumer interest while addressing grower needs.

## Conclusion

This work adds to the currently limited pool of resources available for dry bean breeders to target fast cooking time, flavor, and texture in their breeding programs. The QTL identified in this work, in particular CT8.2 and CT10.2, can be used to develop molecular markers for the incorporation of fast cooking time into new bean varieties to benefit both consumers and the canning industry. For sensory attributes, many QTL for attribute intensities including total flavor, beany, earthy, starchy, sweet, bitter, seed-coat perception, and cotyledon texture were consistent across years and show potential for use in marker-assisted selection following identification of breeding targets informed by consumer preference. Consumers are seeking pulse products with improved culinary characteristics and unique appearance. Yellow dry beans like those used in this study are unfamiliar to United States consumers, but they tend to be fast cooking with desirable sensory attributes. With the recent increased interest in plant-based proteins, now is an opportune time to address consumer preference in dry beans to remain competitive with other pulses, and yellow beans might be an ideal vehicle to a fast-cooking, flavorful, and flourishing future of dry beans.

## Data Availability Statement

The phenotypic data, genotypic data, linkage map, and QTL results relevant for this study are provided as Supplementary Materials.

## Ethics Statement

The studies involving human participants were reviewed and approved by the Institutional Review Board at Michigan State University (IRB# x16-763e Category: Exempt 6). The patients/participants provided their written informed consent to participate in this study.

## Consent to Participate

Informed consent was obtained from all panelists.

## Author Contributions

AB contributed to methodology design, collected, analyzed, and visualized the data, and wrote the manuscript. DK developed the RIL population, collected leaf tissue for DNA extraction, and edited the manuscript. QS genotyped the RIL population and edited the manuscript. KC conceptualized the study, acquired the funding, designed field trials, and contributed to writing and editing the manuscript. All authors contributed to the article and approved the submitted version.

## Conflict of Interest

The authors declare that the research was conducted in the absence of any commercial or financial relationships that could be construed as a potential conflict of interest.

## Footnotes

1 http://phytozome.jgi.doe.gov/
