# Supplementary material for: QTL Mapping of Seed Quality Traits Including Cooking Time, Flavor, and Texture in a Yellow Dry Bean (Phaseolus vulgaris L.) Population
Source: Front Plant Sci. 2021 Jun 22;12:670284. doi: 10.3389/fpls.2021.670284 (PMC8259628; doi:10.3389/fpls.2021.670284)
Supplement: Supplementary file 1 [file Data_Sheet_1.PDF]

QTL mapping of seed quality traits including cooking time, flavor, and texture in a yellow dry bean (*Phaseolus vulgaris* L.) population

Amber Bassett[1], Dennis N. Katuuramu[12], Qijian Song[3], Karen Cichy[14]*

[1] Department of Plant, Soil and Microbial Sciences, Michigan State University, East Lansing, MI
[2] U.S. Vegetable Laboratory, USDA-ARS, Charleston, SC
[3] Beltsville Agricultural Research Center, USDA-ARS, Beltsville, MD
[4] Sugarbeet and Bean Research Unit, USDA-ARS, East Lansing, MI

*Corresponding Author: Phone: 517-353-0210; Email: karen.cichy@usda.gov

Supplementary Figures and Tables

Figure S1 Density plots of seed weight and seed yield for the RILs from 2016, 2017, and both years combined (C). Means for Ervilha and PI527538 from both years combined (2017 for seed yield) are indicated in yellow and brown, respectively

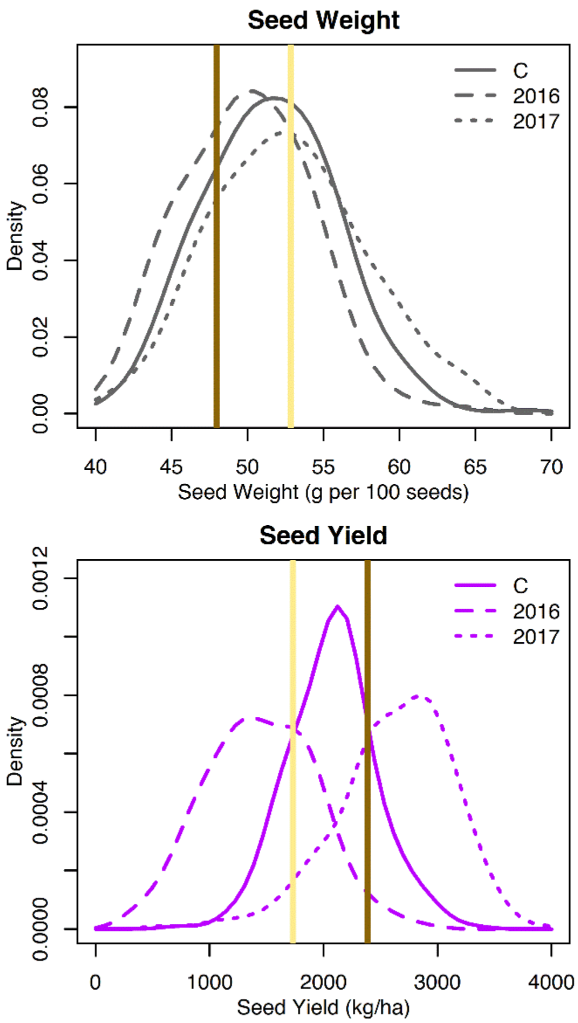

Figure S2 QTL map for seed weight (SW) and seed yield (SY) in the RIL population. Size is in cM. Year is indicated for each QTL, where "C" is both years combined

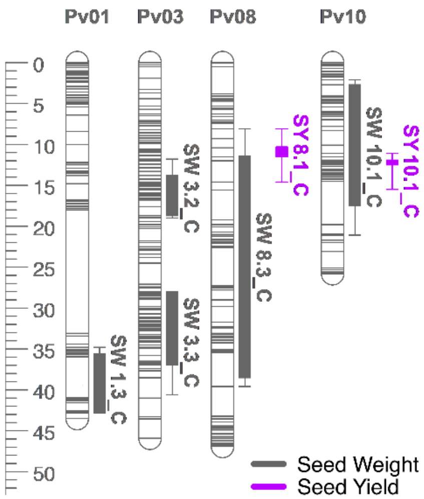

Table S1 Parental phenotypes, means, ranges, and broad-sense heritability (H^2) for the RILs for both years combined with ANOVA *p*-values for genotype, year, and genotype by year indicated.

Trait	Ervilha	PI527538	Mean[a]	Range	H^2	Genotype	Year	Genotype x Year
Seed Weight (g/100 seeds)								
	52.8[a] ± 0.1	48.0[b] ± 1.2	51.35 ± 0.3	39.1 - 68.4	0.84	< 0.0001	< 0.0001	< 0.0001
Seed Yield (kg/ha)								
	1891.6[a] ± 403.9	1731.4[a] ± 639.2	2072.9 ± 23.8	751.0 – 3283.9	0.57	< 0.0001	< 0.0001	< 0.0001

[a] Mean separation is indicated by letter superscript.

Table S2 Parental phenotypes and means and ranges for the RILs for 2016 and 2017.

Trait	Year	Ervilha	PI527538	Mean	Range
Water Uptake (%)					
	2016	104.3 ± 1.9	97.0 ± 0.1	99.0 ± 0.5	38.6 - 114.9
	2017	114.2 ± 4.8	100.5 ± 4.8	103.9 ± 0.3	90.2 - 135.7
Cooking Time (min)					
	2016	23.1 ± 0.6	33.2 ± 1.4	27.2 ± 0.3	19.7 - 40.3
	2017	18.8 ± 0.3	26.3 ± 1.1	23.6 ± 0.2	17.8 - 33.0
Total Flavor Intensity (0-5)					
	2016	3.2 ± 0.1	3.4 ± 0.1	3.4 ± 0.0	2.1 - 4.2
	2017	2.9 ± 0.0	3.1 ± 0.0	3.2 ± 0.0	2.1 - 4.2
Beany Flavor Intensity (0-5)					
	2016	2.4 ± 0.2	3.5 ± 0.1	3.0 ± 0.0	1.8 - 4.1
	2017	1.9 ± 0.1	3.1 ± 0.1	3.0 ± 0.0	1.5 - 3.9
Vegetative Flavor Intensity (0-5)					
	2016	2.9 ± 0.1	2.6 ± 0.0	2.7 ± 0.0	1.7 - 3.9
	2017	2.5 ± 0.2	2.4 ± 0.2	2.5 ± 0.0	1.5 - 3.8
Earthy Flavor Intensity (0-5)					
	2016	2.0 ± 0.0	2.2 ± 0.1	2.3 ± 0.0	1.2 - 4.0
	2017	2.0 ± 0.0	2.2 ± 0.0	2.2 ± 0.0	1.3 - 3.0
Starchy Flavor Intensity (0-5)					
	2016	3.6 ± 0.1	3.1 ± 0.0	3.2 ± 0.0	2.5 - 4.1
	2017	3.6 ± 0.0	2.9 ± 0.1	3.1 ± 0.0	2.2 - 4.0
Sweet Flavor Intensity (0-5)					
	2016	2.2 ± 0.2	1.7 ± 0.1	2.0 ± 0.0	1.1 - 3.1
	2017	2.5 ± 0.0	1.9 ± 0.1	2.1 ± 0.0	1.2 - 3.1
Bitter Flavor Intensity (0-5)					
	2016	1.4 ± 0.1	2.0 ± 0.1	1.7 ± 0.0	0.9 - 2.5
	2017	1.3 ± 0.0	1.8 ± 0.0	1.7 ± 0.0	0.9 - 2.8
Seed-coat Perception (0-5)					
	2016	2.9 ± 0.0	3.4 ± 0.1	3.1 ± 0.0	2.3 - 3.9
	2017	2.6 ± 0.0	3.4 ± 0.1	3.0 ± 0.0	2.0 4.1
Cotyledon Texture (0-5)					
	2016	2.3 ± 0.0	1.8 ± 0.1	2.3 ± 0.0	1.4 - 3.3
	2017	2.5 ± 0.2	2.2 ± 0.0	2.3 ± 0.0	1.4 - 3.3
L*					
	2016	65.2	51.6	59.9 ± 0.3	49.3 - 68.0
	2017	64.5	56.6	58.0 ± 0.3	40.3 - 68.8
a*					
	2016	-1.5	3.7	1.4 ± 0.1	-3.2 - 5.9
	2017	0.1	3.2	1.1 ± 0.1	-2.4 - 4.7

Trait	Year	Ervilha	PI527538	Mean	Range
b*					
	2016	21.0	11.4	19.9 ± 0.3	8.4 - 34.4
	2017	23.5	17.8	23.6 ± 0.2	12.8 - 34.6
Seed-coat Postharvest Non-darkening (0 = Non-darkening; 1 = Darkening)					
	2016	.	.	.	.
	2017	0	1	0.5 - 0.0	0.0 - 1.0
Seed Weight (g/100 seeds)					
	2016	53.0 ± 1.8	46.3 ± 1.4	49.9 ± 0.3	39.5 - 64.7
	2017	52.7 ± 2.3	49.7 ± 0.9	52.7 ± 0.3	39.1 - 72.1
Seed Yield (kg/ha)					
	2016	.	1399.2 ± 23.1	1448.3 ± 32.0	301.0 - 2876.5
	2017	1731.5 ± 639.2	2384.4 ± 591.0	2623.2 ± 32.4	592.1 - 3912.0

Table S3 *P*-values for the random effects from the sensory attribute intensity ANOVAs at the genotype level.

Trait	Rep	Panelist(Year)	Session(Year)
Total Flavor Intensity	NS	<0.0001	<0.0001
Beany Intensity	NS	<0.0001	0.0145
Vegetative Intensity	NS	<0.0001	<0.0001
Earthy Intensity	NS	<0.0001	NS
Starchy Intensity	NS	<0.0001	0.0136
Sweet Intensity	NS	<0.0001	<0.0001
Bitter Intensity	NS	<0.0001	0.005
Seed-coat Perception	NS	<0.0001	<0.0001
Cotyledon Texture	NS	<0.0001	<0.0001

NS indicates non-significant *p*-values at $\alpha = 0.05$

Table S4 Quantitative trait loci identified in the RIL population (N = 240) grown in Entrican, MI in 2016 and 2017 for water uptake and cooking time. Linkage group (LG), year, peak position (Pos), logarithm of odds (LOD), R^2, QTL effect (a), physical interval, map interval, and significance of the QTL are indicated.

Trait	QTL Name	LG	Year[a]	Pos (bp)	Pos (cM)	LOD	R^2 (%)	a[b]	Physical Interval[c] (Mb)	Map Interval[d] (cM)	Sig[e]
Water Uptake											
	WU4.1	Pv04	C	53507	0.01	2.8	4.1	-	0.05 - 0.24	0.01 - 0.50	**
	WU4.2	Pv04	2017	45728204	32.55	3.6	6.2	+	45.04 - 47.61	30.16 - 35.55	**
	WU9.1	Pv09	C	13800234	10.76	3.7	5.7	+	12.60 - 16.11	8.46 - 13.66	**
		Pv09	2016	14837959	12.53	2.9	4.3	+	13.67 - 15.95	10.57 - 13.38	**
		Pv09	2017	13800234	10.76	3.3	4.9	+	13.67 - 13.85	10.57 - 10.85	**
	WU10.1	Pv10	C	41060322	11.99	5.5	9.2	+	3.45 - 42.26	1.83 - 13.77	**
		Pv10	2016	41093812	12.08	6.2	10.1	+	3.45 - 42.26	1.83 - 13.77	**
		Pv10	2017	39257562	7.82	4.6	6.9	+	0.29 - 42.65	0.01 - 14.34	**
Cooking Time											
	CT2.2	Pv02	C	44116993	40.44	3.6	5.6	-	42.75 - 44.12	39.44 - 41.82	**
		Pv02	2016	44116993	40.44	3.4	7.1	-	42.75 - 44.12	39.44 - 41.44	**
	CT2.3	Pv02	C	47982820	50.60	3.6	4.5	-	46.45 - 48.35	47.31 - 50.69	**
		Pv02	2016	46879774	47.50	3.5	6.0	-	45.15 - 48.35	43.74 - 50.69	**
	CT5.3	Pv05	C	40651928	35.04	3.7	4.7	-	40.52 - 40.68	34.76 - 35.04	**
		Pv05	2016	40651928	35.04	2.7	4.7	-	40.52 - 40.68	34.76 - 35.04	*
	CT8.1	Pv08	C	1835444	9.12	2.8	3.6	-	1.26 - 2.17	7.35 - 9.29	*
		Pv08	2017	1280867	7.45	6.0	6.5	-	0.89 - 4.00	5.73 - 14.05	**
	CT8.2	Pv08	C	60515678	36.45	7.5	10.2	+	53.03 - 62.50	25.56 - 44.90	**
		Pv08	2016	61230243	38.45	6.1	11.6	+	53.03 - 62.14	25.56 - 43.58	**
		Pv08	2017	60515678	35.45	7.9	8.7	+	53.03 - 62.58	25.56 - 45.37	**
	CT10.2	Pv10	C	41060322	11.99	15.6	22.3	-	37.83 - 43.84	6.17 - 21.13	**
		Pv10	2016	41060322	11.99	16.6	23.8	-	37.57 - 43.84	5.98 - 21.13	**
		Pv10	2017	41060322	11.99	21.7	27.6	-	37.57 - 43.84	5.98 - 21.13	**

The largest LOD and R^2 within the QTL are reported

[a] "C" indicates both years combined

[b] + and – indicate positive and negative effects on the mean as conferred by alleles from Ervilha in the QTL region.

[c] Physical positions of the nearest markers upstream and downstream of the map interval

[d] Region where LOD scores are significant at the indicated significance level

[e] Significance at $\alpha = 0.1$ and $\alpha = 0.05$ are indicated by * and **, respectively, based on 1000 permutations

Table S5 Quantitative trait loci identified in the RIL population (N = 240) grown in Entrican, MI in 2016 and 2017 for sensory attributes. Linkage group (LG), year, peak position (Pos), logarithm of odds (LOD), R^2, QTL effect (a), physical interval, map interval, and significance of the QTL are indicated.

Trait	QTL Name	LG	Year[a]	Pos (bp)	Pos (cM)	LOD	R^2 (%)	a[b]	Physical Interval[c] (Mb)	Map Interval[d] (cM)	Sig[e]
Total Flavor Intensity											
	TFI2.1	Pv02	C	31802612	27.92	3.1	3.9	-	31.80 - 33.08	27.92 - 28.90	**
	TFI3.1	Pv03	C	41406149	27.09	5.0	6.1	-	4.16 - 43.22	13.80 - 28.25	**
		Pv03	2016	41406149	27.09	3.8	5.1	-	39.67 - 42.36	25.55 - 27.48	**
		Pv03	2017	33779249	20.15	4.1	6	-	29.62 - 41.84	18.61 - 27.09	**
	TFI7.1	Pv07	C	6719315	17.15	8.1	10.9	+	3.79 - 34.27	14.77 - 25.45	**
		Pv07	2016	6293696	16.96	8.6	12.9	+	1.62 - 34.27	13.11 - 25.45	**
		Pv07	2017	6719315	17.15	3.1	4.3	+	5.45 - 7.40	16.58 - 17.44	**
	TFI9.1	Pv09	2016	16265428	17.51	3.0	4.8	+	16.27 - 31.78	16.51 - 22.53	**
	TFI10.1	Pv10	C	41060322	11.99	13.2	18.4	-	38.84 - 43.47	7.03 - 18.53	**
		Pv10	2016	41060322	11.99	8.0	11.3	-	39.72 - 43.47	9.30 - 16.53	**
		Pv10	2017	41182662	12.18	9.6	14.8	-	39.72 - 43.47	9.30 - 17.53	**
Beany Intensity											
	BFI3.1	Pv03	C	32070949	18.90	11.0	13.1	-	5.95 - 48.71	15.12 - 32.83	**
		Pv03	2016	32070949	18.90	8.1	10.6	-	4.16 - 42.36	13.80 - 27.48	**
		Pv03	2017	32803416	19.00	12.8	16.3	-	3.29 - 44.83	11.34 - 28.91	**
	BFI10.1	Pv10	C	41060322	11.99	18.8	25.1	-	3.45 - 43.85	1.83 - 21.71	**
		Pv10	2016	40959742	11.90	15.9	22.5	-	22.43 - 43.84	4.55 - 21.13	**
		Pv10	2017	41182662	12.18	15.4	18.9	-	7.46 - 43.47	4.07 - 19.53	**
Vegetative Intensity											
	VFI7.1	Pv07	C	6719315	17.15	5.8	9.3	+	1.62 - 36.08	13.11 - 28.30	**
		Pv07	2016	6604875	17.01	6.6	11.4	+	1.62 - 36.08	13.11 - 28.30	**
Earthy Intensity											
	EFI1.1	Pv01	2017	5533361	12.91	3.5	5.8	-	5.53 - 5.87	12.91 - 13.21	**
	EFI3.1	Pv03	2016	39674302	24.55	4.3	8.6	-	39.29 - 41.41	23.77 - 26.55	**
		Pv03	2017	38958039	23.38	3.9	6.5	-	33.07 - 39.67	19.38 - 23.77	**
	EFI3.2	Pv03	C	48019631	32.64	3.8	5.9	-	46.42 - 49.58	31.48 - 34.77	**
		Pv03	2016	49321493	34.18	3.5	6.4	-	48.92 - 49.44	33.51 - 34.18	**
Starchy Intensity											
	STI1.1	Pv01	C	51140837	42.57	3.4	4.6	+	50.95 - 51.19	42.57 - 42.65	**
	STI3.1	Pv03	C	30678469	18.71	8.9	12.6	+	3.76 - 43.47	12.37 - 28.44	**
		Pv03	2016	5950105	15.12	3.4	5.6	+	5.01 - 32.07	14.75 - 18.71	**
		Pv03	2017	36758898	21.34	10.1	15.6	+	4.16 - 43.22	13.80 - 28.25	**
	STI6.1	Pv06	C	30971026	29.77	3.7	5.1	+	30.66 - 30.97	28.19 - 29.77	**
		Pv06	2017	30766445	28.77	3.6	5.9	+	30.77 - 30.97	28.77 - 29.77	**
	STI8.1	Pv08	2016	6096106	19.25	3.4	5.6	+	4.41 - 6.80	18.64 - 19.25	**
	STI10.1	Pv10	C	42224711	13.77	5.1	6.9	+	40.90 - 43.47	11.51 - 14.53	**
		Pv10	2016	42224711	13.77	3.2	5.3	+	42.22 - 42.32	13.77 - 14.05	**
		Pv10	2017	41402206	12.55	2.7	4.5	+	41.31 - 41.51	12.46 - 12.55	*
Sweet Intensity											
	SWI1.1	Pv01	C	50948757	41.57	2.7	3.8	+	50.95 - 51.14	41.57 - 42.57	*
		Pv01	2016	50948757	41.57	3.4	5.0	+	50.88 - 51.14	41.20 - 41.57	**
	SWI2.1	Pv02	C	25666553	22.99	3.0	4.1	+	25.67 - 27.31	22.99 - 23.18	**
	SWI3.1	Pv03	C	29623010	17.61	3.8	5.4	+	29.62 - 30.68	17.61 - 18.61	**
		Pv03	2016	29623010	17.61	3.5	5.3	+	29.62 - 32.07	17.61 - 18.71	**
	SWI6.1	Pv06	2017	30617829	27.63	3.2	4.9	+	30.10 - 30.77	27.63 - 28.19	**

Trait	QTL Name	LG	Year[a]	Pos (bp)	Pos (cM)	LOD	R^2 (%)	a[b]	Physical Interval[c] (Mb)	Map Interval[d] (cM)	Sig[e]
	SWI7.1	Pv07	C	29986177	21.23	6.2	9.9	+	8.02 - 34.78	17.82 - 26.72	**
		Pv07	2016	29986177	21.23	5.4	9.2	+	6.29 - 34.27	16.96 - 24.45	**
		Pv07	2017	30201831	22.60	2.9	4.3	+	10.33 - 30.62	22.23 - 22.88	*
	SWI8.1	Pv08	C	2171710	10.37	5.1	5.4	+	0.13 - 4.00	1.49 - 13.05	**
		Pv08	2016	556924	2.49	3.9	6.6	+	0.13 - 0.81	1.49 - 4.68	**
		Pv08	2017	2658499	11.47	4.3	6.6	+	1.28 - 4.00	7.45 - 13.05	**
	SWI8.2	Pv08	C	6802573	19.82	3.0	3.9	+	6.80 - 7.97	19.82 - 20.10	**
		Pv08	2016	6802573	19.82	3.5	5.2	+	6.80 - 7.97	19.82 - 20.10	**
		Pv08	2017	9350287	21.24	3.0	4.6	+	9.35 - 13.73	21.24 - 21.62	**
Bitter Intensity											
	BI2.1	Pv02	C	27306217	23.66	3.1	5.0	-	27.31 - 28.36	23.66 - 23.75	**
		Pv02	2017	27578608	23.75	3.1	4.5	-	27.58 - 31.80	23.75 - 24.53	**
	BI3.1	Pv03	C	32070949	18.90	7.4	10.6	-	3.84 - 42.96	12.85 - 28.15	**
		Pv03	2016	28686655	16.82	5.0	8.5	-	3.76 - 36.76	12.37 - 20.34	**
		Pv03	2017	32803416	19.00	7.0	10.5	-	5.00 - 42.36	14.65 - 27.48	**
	BI10.1	Pv10	C	40959742	11.90	5.4	8.7	-	39.72 - 43.47	8.30 - 17.53	**
		Pv10	2017	40959742	11.90	4.5	6.6	-	40.84 - 41.6	11.13 - 12.74	**
Seed-coat Perception											
	SPE1.1	Pv01	C	51140837	42.57	4.1	5.7	-	50.83 - 51.33	40.92 - 42.84	**
		Pv01	2017	50906825	41.29	4.4	6.2	-	49.66 - 51.33	38.97 - 42.84	**
	SPE3.1	Pv03	C	32070949	18.90	13.3	20.1	-	3.00 - 41.41	10.59 - 26.55	**
		Pv03	2016	30678469	18.61	5.3	9.3	-	5.95 - 36.76	15.12 - 20.34	**
		Pv03	2017	29623010	17.61	11.2	17.1	-	3.00 - 41.41	10.59 - 25.55	**
	SPE10.1	Pv10	C	40959742	11.90	2.8	4.0	-	40.96 - 42.26	11.90 - 13.77	*
		Pv10	2017	42167184	13.58	3.4	4.9	-	40.96 - 42.26	11.90 - 13.77	**
Cotyledon Texture											
	CTX3.1	Pv03	C	32803416	19.00	2.8	4.1	+	32.07 - 33.78	18.90 - 19.76	*
		Pv03	2016	32803416	19.00	3.8	6.1	+	7.29 - 36.76	16.07 - 21.34	**
	CTX4.1	Pv04	C	7368228	18.23	3.1	4.7	+	7.37 - 9.45	18.23 - 18.71	**
		Pv04	2016	7568594	18.52	3.0	4.9	+	7.57 - 9.45	18.52 - 18.71	**
	CTX7.1	Pv07	C	8690008	18.10	4.1	6.0	+	5.45 - 29.99	16.58 - 21.23	**
		Pv07	2016	4183079	15.35	3.5	5.5	+	3.74 - 6.06	14.48 - 16.77	**
	CTX7.2	Pv07	2017	34576426	26.72	3.4	5.7	+	34.27 - 36.08	26.43 - 28.30	**
	CTX10.1	Pv10	C	42451057	14.34	6.4	9.6	+	39.72 - 43.47	9.30 - 18.53	**
		Pv10	2016	42224711	13.77	3.5	5.5	+	40.96 - 42.65	11.90 - 14.34	**

The largest LOD and R^2 within the QTL are reported

[a] "C" indicates both years combined

[b] + and – indicate positive and negative effects on the mean as conferred by alleles from Ervilha in the QTL region.

[c] Physical positions of the nearest markers upstream and downstream of the map interval

[d] Region where LOD scores are significant at the indicated significance level

[e] Significance at $\alpha = 0.1$ and $\alpha = 0.05$ are indicated by * and **, respectively, based on 1000 permutations

Table S6 Quantitative trait loci identified in the RIL population (N = 240) grown in Entrican, MI in 2017 for color and seed-coat postharvest non-darkening. Linkage group (LG), year, peak position (Pos), logarithm of odds (LOD), R^2, QTL effect (a), physical interval, map interval, and significance of the QTL are indicated.

Trait	QTL Name	LG	Year[a]	Pos (bp)	Pos (cM)	LOD	R^2 (%)	a[b]	Physical Interval[c] (Mb)	Map Interval[d] (cM)	Sig[e]
L*											
	SL*3.1	Pv03	C	39674302	25.6	7.8	4.5	+	3.91 - 48.14	13.32 - 32.64	**
		Pv03	2016	39674302	25.6	7.3	4.3	+	3.91 - 48.14	13.32 - 32.64	**
		Pv03	2017	39287195	23.8	5.3	4.2	+	29.62 - 42.36	18.61 - 27.48	**
	SL*6.1	Pv06	C	17329576	4.9	8.2	4.5	+	6.42 - 20.51	0.01 - 9.19	**
		Pv06	2016	17329576	4.9	8.2	4.7	+	6.42 - 20.51	0.01 - 9.64	**
		Pv06	2017	17329576	4.6	6.5	5.4	+	6.42 - 20.51	0.01 - 8.19	**
	SL*8.1	Pv08	C	3007875	12.1	10.4	5.8	+	0.63 - 24.47	4.11 - 22.00	**
		Pv08	2016	3007875	12.1	10.4	6.1	+	0.13 - 24.47	3.49 - 22.00	**
		Pv08	2017	3007875	12.1	8.6	7.1	+	0.63 - 4.41	4.11 - 14.56	**
	SL*10.1	Pv10	C	40959742	11.9	60.0	55.6	+	3.45 - 43.85	1.83 - 21.71	**
		Pv10	2016	40959742	11.9	58.1	54.5	+	3.45 - 43.85	1.83 - 21.71	**
		Pv10	2017	40959742	11.9	36.8	40.0	+	3.45 - 43.85	1.83 - 21.71	**
a*											
	Sa*1.1	Pv01	C	1333499	2.2	3.2	2.4	+	1.33 - 1.42	2.21 - 2.31	**
		Pv01	2016	1333499	2.2	3.2	2.5	+	1.33 - 1.42	2.21 - 2.31	**
	Sa*3.1	Pv03	C	43470352	28.5	6.0	4.4	-	3.16 - 48.92	10.96 - 33.41	**
		Pv03	2016	43712075	28.8	5.9	4.3	-	3.16 - 48.92	10.96 - 33.41	**
		Pv03	2017	39674302	24.6	6.7	5.7	-	5.00 - 48.14	14.65 - 32.64	**
	Sa*3.2	Pv03	C	51330379	38.5	3.3	2.5	-	51.33 - 52.15	38.48 - 38.58	**
		Pv03	2016	51330379	38.5	3.4	2.6	-	51.33 - 52.15	38.48 - 38.58	**
	Sa*10.1	Pv10	C	40959742	11.9	44.9	51.9	-	3.45 - 43.85	1.83 - 21.71	**
		Pv10	2016	40959742	11.9	44.6	51.7	-	3.45 - 43.85	1.83 - 21.71	**
		Pv10	2017	40959742	11.9	40.1	47.7	-	3.45 - 43.85	1.83 - 21.71	**
b*											
	Sb*3.1	Pv03	C	39287195	23.8	4.7	5.9	+	29.62 - 41.84	17.61 - 27.09	**
		Pv03	2016	39287195	23.8	4.4	5.5	+	29.62 - 41.84	17.61 - 27.09	**
		Pv03	2017	29623010	17.6	3.2	4.1	+	29.62 - 30.68	17.61 - 18.61	**
	Sb*4.1	Pv04	C	421790	0.8	3.4	4.2	+	0.13 - 2.06	0.50 - 6.02	**
		Pv04	2016	421790	0.8	3.3	4.4	+	0.13 - 2.06	0.50 - 6.02	**
	Sb*4.2	Pv04	C	45728204	34.6	4.7	8.3	+	45.04 - 47.65	29.16 - 39.6	**
		Pv04	2016	47614667	35.6	3.6	6.8	+	45.73 - 47.61	32.55 - 38.55	**
		Pv04	2017	45728204	34.6	3.0	4.8	+	45.73 - 47.61	32.55 - 36.55	*
	Sb*5.1	Pv05	C	219601	0.0	3.0	3.7	-	0.22 - 0.52	0.01 - 1.01	**
	Sb*10.1	Pv10	C	41510878	12.7	12.7	17.2	+	37.83 - 43.47	6.17 - 18.53	**
		Pv10	2016	41060322	12.0	13.4	19.9	+	37.57 - 43.47	5.98 - 18.53	**
		Pv10	2017	41510878	12.7	17.5	23.7	+	38.27 - 43.47	6.46 - 18.53	**
Seed-coat Postharvest Non-darkening											
	ND10.1[WP,YY]	Pv10	2017	43465901	18.5	81.2[f]	87.5	-	3.98 - 43.85	2.11 - 21.71	**

The largest LOD and R^2 within the QTL are reported

[a] "C" indicates both years combined

[b] + and – indicate positive and negative effects on the mean as conferred by alleles from Ervilha in the QTL region.

[c] Physical positions of the nearest markers upstream and downstream of the map interval

[d] Region where LOD scores are significant at the indicated significance level

[e] Significance at $\alpha = 0.1$ and $\alpha = 0.05$ are indicated by * and **, respectively, based on 1000 permutations

[f] Many LODs in the map interval for ND.10.1 were not able to be reported by QTL Cartographer so the highest reported LOD is indicated

Table S7 Quantitative trait loci identified in the RIL population (N = 240) grown in Entrican, MI in 2016 and 2017 for seed weight and seed yield. Linkage group (LG), year, peak position (Pos), logarithm of odds (LOD), R^2, QTL effect (a), physical interval, map interval, and significance of the QTL are indicated.

Trait	QTL Name	LG	Year[a]	Pos (bp)	Pos (cM)	LOD	R^2 (%)	a[b]	Physical Interval[c] (Mb)	Map Interval[d] (cM)	Sig[e]
Seed Weight											
	SW1.3	Pv01	C	51192287	42.84	6.1	6.7	+	49.57 - 51.33	35.59 - 42.84	**
		Pv01	2016	51192287	42.84	5.7	6.0	+	49.66 - 51.33	36.97 - 42.84	**
		Pv01	2017	51140837	42.65	4.6	4.9	+	50.95 - 51.33	41.57 - 42.84	**
	SW3.2	Pv03	C	29623010	17.61	4.3	4.8	+	4.16 - 32.07	13.80 - 18.71	**
		Pv03	2016	28686655	16.82	7.5	8.2	+	3.00 - 36.76	10.59 - 21.34	**
		Pv03	2017	28686655	16.82	3.8	3.7	+	6.54 - 30.68	15.59 - 17.61	**
	SW3.3	Pv03	C	43547357	28.72	7.1	6.9	-	42.36 - 50.66	27.96 - 36.94	**
		Pv03	2016	43547357	28.72	8.2	8.8	-	41.57 - 50.30	27.09 - 36.56	**
		Pv03	2017	47004660	32.06	3.5	3.5	-	47.09 - 49.97	32.06 - 34.87	**
	SW6.2	Pv06	2016	30766445	28.77	3.1	3.1	+	30.77 - 30.97	28.77 - 29.77	**
		Pv06	2017	30617829	27.91	5.2	5.5	+	29.85 - 30.97	25.69 - 29.77	**
	SW8.3	Pv08	C	53028165	24.56	14.0	22.0	+	2.17 - 61.23	11.37 - 38.45	**
		Pv08	2016	6096106	19.25	17.3	20.6	+	1.84 - 61.23	10.29 - 37.45	**
		Pv08	2017	53028165	24.56	13.8	21.0	+	4.41 - 61.23	16.64 - 39.45	**
	SW10.1	Pv10	C	40839326	11.13	6.0	6.7	-	5.73 - 43.47	2.69 - 17.53	**
		Pv10	2016	41060322	11.99	4.6	4.8	-	39.72 - 42.21	9.30 - 13.67	**
		Pv10	2017	41060322	11.99	7.5	8.2	-	5.73 - 43.47	3.69 - 18.53	**
Seed Yield											
	SY3.4	Pv03	2016	4162402	13.80	3.4	4.9	+	4.16 - 4.47	13.80 - 14.18	**
	SY4.1	Pv04	2017	47614667	39.60	4.5	7.5	-	45.73 - 48.01	35.55 - 40.74	**
	SY8.1	Pv08	C	2658499	11.37	3.2	5.2	+	1.84 - 3.01	10.29 - 11.47	**
		Pv08	2016	2171710	10.37	5.9	8.7	+	0.87 - 4.00	5.64 - 13.05	**
	SY10.1[MS,YY]	Pv10	C	40959742	11.90	3.6	5.8	-	40.96 - 41.40	11.90 - 12.46	**
		Pv10	2016	41510878	12.74	8.2	12.6	-	40.84 - 43.47	11.13 - 17.53	**
	SY11.2	Pv11	2016	1000205	1.98	3.5	5.6	+	0.88 - 3.21	0.40 - 4.98	**

The largest LOD and R^2 within the QTL are reported

[a] "C" indicates both years combined

[b] + and – indicate positive and negative effects on the mean as conferred by alleles from Ervilha in the QTL region.

[c] Physical positions of the nearest markers upstream and downstream of the map interval

[d] Region where LOD scores are significant at the indicated significance level

[e] Significance at $\alpha = 0.1$ and $\alpha = 0.05$ are indicated by * and **, respectively, based on 1000 permutations

SY10.1 was previously detected via joint QTL analysis of the Merlot x S48M, S94M, and S95M populations (Hoyos-Villegas, V., Song, Q., Wright, E.M., Beebe, S.E., and Kelly, J.D. (2016). Joint linkage QTL mapping for yield and agronomic traits in a composite map of three common beans RIL populations. *Crop Sci*. 56, 2546-2563. **https://doi.org/10.2135/cropsci2016.01.0063**)
